# 10 Years of BRAVE Self-Help Service Delivery for Child and Adolescent Anxiety in Australia: Open Effectiveness-Implementation Trial

**DOI:** 10.2196/97810

**Published:** 2026-10-05

**Authors:** Sonja March, Alimila Hayixibayi, Arlen Kate Rowe, Jay Michel Stevens, Kirsty Zieschank, Caroline Leanne Donovan, Susan H Spence

**Affiliations:** 1 Institute for Health, University of Southern Queensland, UniSQ Springfield Campus, 37 Sinnathamby Blvrd, Springfield Central, Queensland, 4300, Australia, 61 734704434; 2 Manna Institute, Toowoomba, Queensland, Australia; 3 School of Health, Psychological and Medical Sciences, University of Southern Queensland, Springfield Central, Queensland, Australia; 4 School of Applied Psychology, Centre for Mental Health, Griffith University, Mount Gravatt, Queensland, Australia; 5 Australian Institute for Suicide Research and Prevention, School of Applied Psychology, Griffith University, Mount Gravatt, Queensland, Australia

**Keywords:** adolescent, anxiety, child, iCBT, internet-based cognitive behavioral therapy, online, implementation-effectiveness

## Abstract

**Background:**

Self-directed digital mental health programs can increase access to evidence-based interventions for child anxiety. However, there has been little comprehensive investigation of real-world dissemination, especially using implementation science approaches.

**Objective:**

This study aimed to describe and comprehensively assess the implementation of the BRAVE Self-Help program for child and adolescent anxiety in Australia over a 10-year period, including implementation strategies during establishment and sustainability phases and implementation-effectiveness outcomes.

**Methods:**

This study was a large, open implementation-effectiveness trial involving Australian child and adolescent participants in the BRAVE Self-Help Program, conducted between January 1, 2015, and December 31, 2024. Implementation strategies were reported descriptively across program establishment and sustainability phases. Implementation and effectiveness outcomes were reported across 10 years, in line with the implementation outcomes framework (IOF) and taxonomy of implementation outcomes for digital interventions. Metrics included adoption, penetration, appropriateness, fidelity, feasibility, acceptability, and effectiveness.

**Results:**

Over the 10-year period, the BRAVE Self-Help program had 53,726 users, including 28,700 children (mean age 9.36, SD 1.40 years) and 25,026 adolescents (mean age 14.15, SD 1.62 years). Data revealed a wide variety of user characteristics in terms of age, gender, geographical location, baseline severity, and referral sources (community, health, and education settings). Improvement in anxiety symptoms from first to last interaction with the program was observed for participants in general, particularly among those with elevated anxiety at program registration (*P*<.001; Cohen *d*=0.54). Of all users completing the registration assessment, 38.72% (20,026/51,721) completed 3 or more of the 10 sessions, with consistent moderate to high satisfaction rates across all sessions.

**Conclusions:**

This study demonstrated that a self-help digital program for child and adolescent anxiety can be successfully disseminated nationally, and that it is feasible, acceptable, and effective for many young people. During a span of 10 years, the BRAVE Self-Help program for child and adolescent anxiety offered evidence-based support to more than 50,000 families. Notably, although progress through sessions was low for some young people, significant improvements could be made in as few as 3 sessions, and decisions to stop treatment occurred for many reasons, including treatment success.

## Introduction

### Background

Anxiety disorders among young people are a significant public health concern, affecting nearly 7% of young people aged 4 to 17 years [1]. In recent years, multiple national and global crises have occurred, including ongoing cost-of-living pressures, climate and weather-related events, and the COVID-19 pandemic, all of which have been associated with increases in mental health problems [2]. One contemporary Australian multiwave cohort study demonstrated that 58% of adolescents reported clinically significant anxiety symptoms at some point during adolescence [3]. Thus, there is an urgent need for scalable solutions that can reach the many young people impacted by anxiety. Open access digital mental health platforms (DMHPs) have been highlighted as a promising way to increase access to evidence-based interventions and represent an approach that is well accepted both by adults [4] and young people [5]. Based on a representative large-scale Australian survey, a higher proportion of adolescents (20.1%) use online mental health programs and resources compared to either conventional health services (18.3%) or school-based supports (2.4%) [6]. DMHPs encompass websites, applications, and other digital systems that deliver guided and unguided mental health assessments and evidence-based interventions, such as cognitive behavioral therapy (CBT) programs. Unlike traditional mental health services that deliver mental health interventions in person (either face-to-face or via telephone), and crisis support services (eg, Lifeline and Kids Helpline) that provide immediate in-person support via telephone or synchronous messaging, DMHPs deliver intervention content digitally, often in self-directed formats via modules and programs. This delivery model, in which the therapeutic content is embedded in a program, can serve as a general avenue for enhanced accessibility to evidence-based interventions; however, the widespread implementation of DMHPs in real-world contexts requires further examination.

### Nationwide Digital Mental Health Programs

Several nationwide DMHPs targeting adults have been implemented in Australia, including THIS WAY UP [7] and MindSpot [4]. MindSpot Clinic is an e-therapy clinic for adults that attracted 121,652 new program users in the first 7 years of clinic operation [4]. Importantly, MindSpot offers assessments and interventions, with an average of 67% of users using the site primarily for assessment and information purposes [4]. Users of this freely available digital program have reported high satisfaction and demonstrated significant improvements in psychological symptoms (eg, anxiety and depression), with large effect sizes [4]. THIS WAY UP offers both guided and unguided programs, with users showing substantial improvements in psychological distress, anxiety, and depression symptoms following online treatment [7,8].

With respect to children, BRAVE-ONLINE is an accessible, evidence-based, and effective DMHP for the treatment of anxiety in Australian children and adolescents. Several randomized controlled trials have demonstrated the efficacy of various BRAVE-ONLINE models of care, including therapist-assisted [9-11], blended care [12], and stepped-care [11,13]. The self-help version of BRAVE-ONLINE (BRAVE Self-Help) was developed in 2014 to deliver a public health model of care aimed at increasing access to evidence-based treatment for child and adolescent anxiety and has been validated in 2 open effectiveness trials [5,14]. In these 2 studies, significant reductions in anxiety were observed for participants who presented with initially elevated levels of anxiety and who engaged with the program [5,14]. Further, March et al [14] demonstrated that significant improvements were made by users presenting with moderate to severe levels of anxiety, although the authors suggested that those with severe anxiety might need additional ongoing support given their average final anxiety score remained in the “elevated” range. Thus, the BRAVE Self-Help program provides an innovative care model with the potential to significantly reduce the national impact of childhood anxiety problems.

### Implementation Science Approaches to DMHPs

Although DMHPs demonstrate promise in handling the ever-increasing demand for mental health services, there is an absence of literature grounded in implementation science frameworks that details the implementation strategies, implementation and effectiveness outcomes, and real-world experience of disseminating them. Implementation science frameworks assist researchers in effectively translating evidence-based interventions into practice and testing them in real-world conditions [15-17]. These include taxonomies that aim to provide recommendations for effective implementation strategies that can be used to enhance adoption, implementation, sustainment, and scale-up of an evidence-based practice, such as Powell et al [18], and taxonomies for assessing implementation outcomes. For example, Proctor et al [19] developed the implementation outcomes framework (IOF), which presents a taxonomy of implementation outcomes to assess implementation success, including acceptability, adoption, appropriateness, feasibility, fidelity, penetration, and sustainability. More recently, efforts have been made to contextualize implementation frameworks and metrics to digital interventions, particularly those that involve minimal provider mediation and greater data collection at the consumer level [20]. Further, the potential of hybrid effectiveness-implementation designs that combine elements of effectiveness and implementation research has highlighted as a strategy for enhancing the translation of research findings into routine adoption [21]. As DMHPs become more embedded in community and routine care settings, there is an urgent need to examine both effectiveness and implementation outcomes to ensure successful translation.

### Study Objectives

Previous studies have examined the feasibility and acceptability of the BRAVE Self-Help program in subsamples of young people using the program. This study aimed to describe the real-world implementation of BRAVE Self-Help via an open effectiveness-implementation trial delivered in a community setting and to comprehensively examine its outcomes over the first 10 years of operation from 2015 to 2024. This study goes beyond prior evaluations of BRAVE Self-Help (focused on establishing initial feasibility and symptom trajectories in subsamples of participants), extends the time frame to 10 years, and examines the implementation outcomes in detail, within a clear implementation science framework. To provide context for the implementation and effectiveness outcomes of the program, the implementation of BRAVE Self-Help was retrospectively documented, including implementation strategies during its establishment and sustainability stages. The extent to which these strategies align with those identified by Powell et al [18] as being important in effective implementation was also examined as a supplementary aim of the study. A detailed description of these implementation activities is provided in Multimedia Appendix 1.

The primary aim of this study was to report on the overall implementation and effectiveness outcomes of BRAVE Self-Help from 2015 to 2024. In doing so, this study drew from Proctor et al’s [19] IOF and Hermes et al’s [20] taxonomy of implementation outcomes for digital interventions with minimal provider mediation. By examining the use and outcomes of the platform over the first 10 years of the program’s implementation, this study aimed to illustrate how a DMHP such as BRAVE Self-Help can provide a viable service delivery approach capable of managing surges in mental health service demands over time. This study will also serve to inform policymakers and health care providers regarding the characteristics of young people accessing DMHPs and will inform the future design and delivery of digital mental health services going forward.

## Methods

### Study Design

This paper reports on an open effectiveness-implementation study intended to evaluate the implementation and effectiveness of the BRAVE Self-Help program when delivered in the community over a 10-year period as an open-access anxiety program for children and adolescents.

### Ethical Considerations

The study received ethical clearance from the 3 main institutions involved in the development and distribution of BRAVE Self-Help – the University of Queensland (2013001397), Griffith University (2020/581), and the University of Southern Queensland (H20REA298).

### Participants and Procedure

Participants included children and adolescents who registered for the publicly available BRAVE Self-Help program for anxiety in Australia. Children and adolescents could self-refer to the program, or be referred by parents, schools, general practitioners, and mental health professionals. The platform is open to families using a computer with an Australian IP address, and can be accessed at any time, from any location. Upon visiting the website, potential users are required to provide informed consent prior to accessing the program. For users aged <16 years, parental consent is also required. Participation in the program is entirely voluntary, and users are informed that they may cease using the program at any time without consequence. Elevated levels of anxiety are not a requirement to engage with the platform; however, for the effectiveness analysis, only users who presented with elevated levels of anxiety were included. Elevated anxiety was classified as users with a score on the Children’s Anxiety Scale 8-item (CAS-8) ≥84^th^ percentile (≥*T* score 60; CAS-8 score ≥10 for boys, ≥11 for other gender, and ≥12 for girls). Access to the program remains open during and after completion of the program, unless the user requests closure of the account.

Participants comprised all children and adolescents registered for the BRAVE Self-Help program from January 1, 2015, to December 31, 2024. Although the program officially launched in mid-2014, the first 6 months constituted a “soft launch” period during which changes were made to processes, the establishment of implementation and refinement of implementation strategies, as well as changes to the data collected. A consistent dataset was in place from January 2015, along with refined implementation strategies for full launch. Thus, January 2015 is considered the active commencement of the program.

### BRAVE Self-Help Intervention

BRAVE Self-Help is an internet-based cognitive behavioral therapy (iCBT) program for child and adolescent anxiety. It includes separate programs for children (7‐12 years) and adolescents (13‐17 years). Child- and adolescent-focused programs include 10 interactive iCBT sessions presented in an engaging manner designed to facilitate motivation and learning. Sessions are completed sequentially, unlocking after each session is completed. Session activities include content delivery (reading and video), skill rehearsal activities, quizzes, and games to consolidate knowledge, and homework tasks to facilitate practice and learning of skills outside the session. Age-appropriate language and graphics are used for each of the programs, as well as age-specific content and examples. Child and adolescent sessions include evidence-based anxiety management strategies such as recognition of physiological anxiety symptoms, relaxation, coping self-talk, cognitive restructuring, and graded exposure. The program tracks the young person’s anxiety symptoms each session and includes clinical tools to assist with CBT exercises (eg, an online tool to develop and implement an exposure hierarchy; a “relaxation room” with resources targeting emotion regulation). For examples of program pages, see Figure 1. For a detailed description of the online program, see previous studies [5,22].

**Figure 1.**
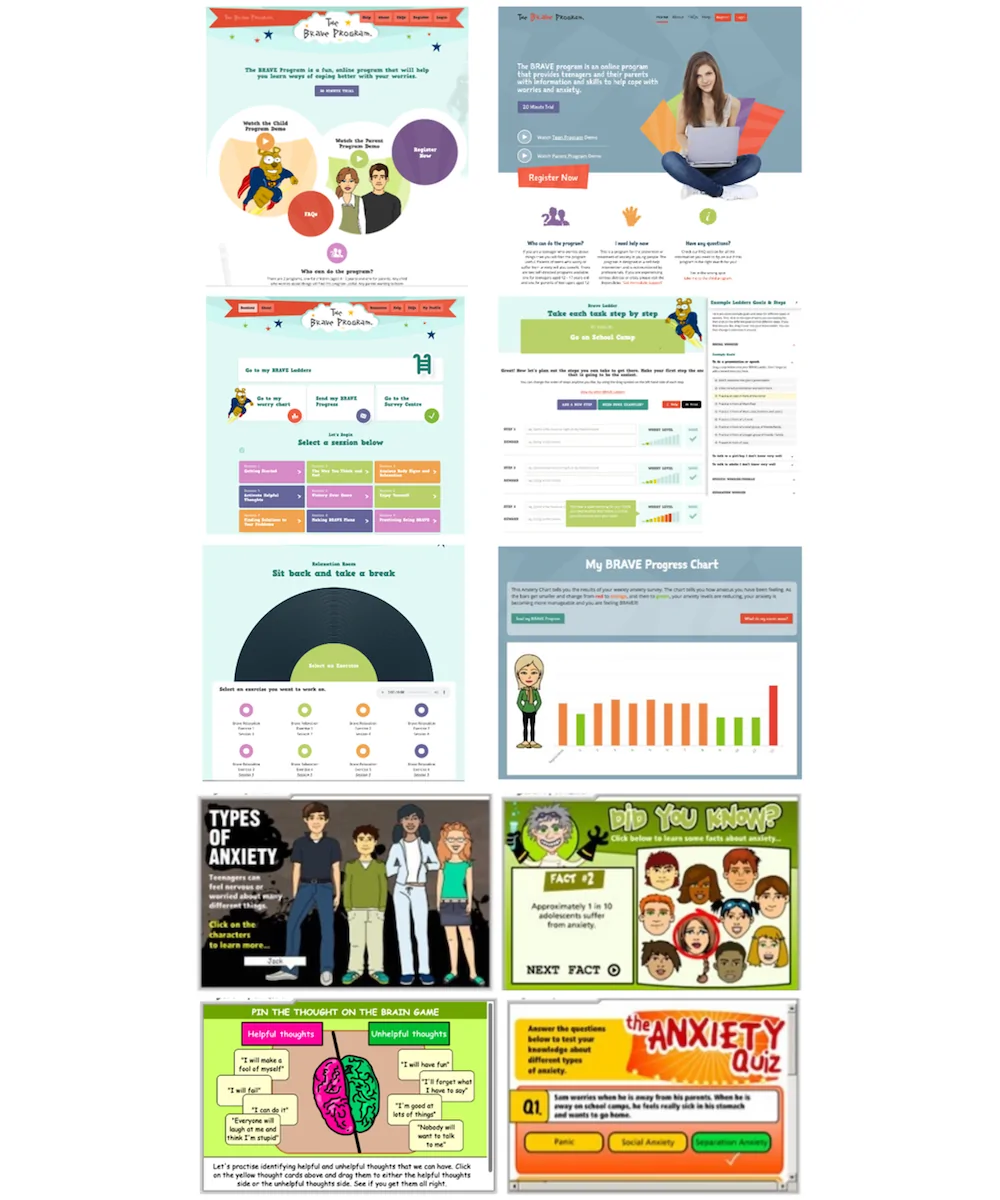
Example pages of the BRAVE Self-Help program.

### Data Collection Procedures and Materials

#### Implementation Context

Implementation strategies were mapped over the establishment (2014‐2016; external funding) and sustainability (2017‐2024; no external funding) phases of the study. Use of implementation strategies was recorded in detail throughout the establishment phase of the project via Microsoft Excel spreadsheets and reports to the funder, and were categorized according to strategy type and targets. Implementation strategies that continued during the sustainability phase were recorded. Strategies were examined retrospectively to assess whether they aligned with the compilation of implementation strategies identified in expert recommendations [18]. Two authors (AH and SM) independently mapped the strategies to expert recommendations, before discussing discrepancies and refining until there was agreement. Strategies were reviewed by a third author (AKR). A detailed summary, including examples of implementation strategies in practice, is presented in Multimedia Appendix 1 to provide the context for the implementation of the DMHP and its outcomes.

#### User Characteristics

##### Socio-Demographics

Basic user demographic information is collected upon registration, including user type (registration in either the child or adolescent program), gender (male, female, or other), age, postcode, and location. Postcode and location were coded in terms of residential remoteness and were classified into major cities and other regional areas, including inner and outer regional, remote, and very remote areas using postcode, based on the Modified Monash Model [23].

##### Referral Sources

Information relating to the referral into the program is collected at registration. Participants are asked to report how they found out about the program (eg, through referral by school or health professional, internet searching), and for those who reported being referred by a health professional, the type of health professional (eg, general practitioner and psychologist) was also reported.

### Effectiveness (Changes in Anxiety Symptom Severity)

Anxiety severity was measured prior to beginning the program at registration, at the commencement of session 1 (if the program was not commenced within 7 days of registration), and at the commencement of each subsequent BRAVE-ONLINE session using the CAS-8 [24]. The CAS-8 includes 8 items that require participants to rate their level of anxiety on a 4-point response scale ranging from 0 (never) to 3 (always). Total scores may range from 0 to 24, with higher total scores indicative of greater levels of anxiety. Example items include “I worry what other people think of me” and “I worry that something bad will happen to me.”

For the purposes of this study, users were categorized into “no elevated anxiety,” “elevated anxiety,” or “clinical anxiety” based on population-level and gender-standardized norms produced via large-scale, school-based prevention program trials [24]. Scores falling <84th percentile (*<T* score 60; CAS-8 score ≤9 for boys and ≤11 for girls) were categorized as “no elevated anxiety,” scores falling ≥84th percentile (≥*T* score 60; CAS-8 score ≥10 for boys, ≥11 for other gender, and ≥12 for girls) were categorized as “elevated anxiety,” and scores ≥94th percentile (*T* score ≥65; CAS-8 score ≥13 for boys, ≥15 for other gender, and ≥16 for girls) were categorized as “clinical anxiety.” The CAS-8 has demonstrated good psychometric properties (Cronbach α=0.89) [24], and for this study, the average internal consistency of the CAS-8 across timepoints was 0.88.

### Program Satisfaction

Program satisfaction is measured via a 5-item scale that gauged the user’s perception of program acceptability at 3 timepoints (after sessions 3, 6, and 9) [5]. For the purposes of this study, the final satisfaction survey the user completed (either after 3, 6, or 9 sessions) was used. Responses are rated on a 5-point scale (1=“definitely not” to 5=“definitely yes”), with total scores ranging from 5 to 25. Higher scores indicate higher levels of satisfaction. Question items include whether users would tell a friend about the program, how helpful they found the program, and how happy they were with the program [5].

### Platform Use

Data relating to use of the program were tracked automatically throughout program participation. The number of new program registrations was captured through total and monthly registrations. The number of users completing the baseline assessment (and therefore able to commence the program) was also calculated. Program completion was operationalized as the number of sessions completed by users (ranging from 0 to 10), with the mean score calculated. The proportion of users completing each session was also calculated.

### Analytic Strategy

To address the primary aim of this study, the implementation and effectiveness outcomes presented in Table 1 (based on Proctor et al’s [19] IOF and Hermes et al’s [20] taxonomy) were analyzed across 10 years. Deidentified data extracted from the BRAVE Self-Help program were visualized using Microsoft Excel Worksheet and Canva and analyzed using SPSS software (version 28.0; IBM Corp). To describe the overall trends over the 10-year period, the number of new registrations per month and year were graphically represented according to school calendar periods, natural disaster events that occurred during each annual period, and the COVID-19 pandemic. School-term dates were compared Australia-wide and holiday periods were mapped for each year. Community- and nationwide natural disaster events were defined as events directly threatening or damaging property or life, with this damage being widespread, and for continued periods, including bushfires, floods, cyclones, supercell storms, heatwaves, and pandemics [25-28]. A negative binomial regression model using a log link function was used to investigate the association of weather events with total monthly registrations while controlling for school holidays, the COVID-19 pandemic, seasonal, and yearly trends.

**Table 1.** Implementation and effectiveness outcomes (mapped to Proctor et al’s [19] and Hermes et al’s [20] frameworks.

Outcomes	Definitions	Metric used in this study
Adoption	Intention, decision, or initiation to use an evidence-based practice	Number of user registrations for the program (behavioral intention to use) Number of assessment completers and program commencements Trends in registrations (monthly and yearly trends)
Penetration	The integration of an evidence-based practice within a service setting and its subsystems	Referral sources (platform reach into services) Location of users (geographical reach) Indigenous status Proportion of user type (child, adolescent, and parent) User characteristics (gender, demographics, and clinical severity)
Fidelity and feasibility	Extent to which implementation results in an evidence-based practice being delivered as intended Extent to which an evidence-based practice can be successfully used or conducted within a given context	User adherence (average number of sessions completed) Program completion (proportion of users completing each session) Reasons for nonadherence User engagement (proportion of users starting, commencing treatment) User adherence (proportion of sessions completed)
Appropriateness and acceptability	Perceived fit, relevance, or compatibility of the evidence-based practice to a given context Perception among users that a given evidence-based practice is useful or satisfactory	Satisfaction with program (mean total satisfaction score) Likelihood to refer program, program helpfulness, happiness with program, helpfulness in feeling less worried, overall satisfaction (mean item scores) Expectations met (proportion)
Effectiveness	Extent to which meaningful changes in target symptoms are made	Changes in anxiety symptom severity (mean changes in Children’s Anxiety Scale) Proportion of users making clinically meaningful change (reductions in clinical severity categories)

In terms of participant characteristics, chi-square analyses were conducted to compare baseline characteristics between program completers (10 sessions) and noncompleters (<10 sessions) and between users who did not start the program and those who completed 1 or more sessions. Additional analyses were conducted to examine the associations between engagement level and baseline characteristics. Finally, a one-way analysis of covariance (ANCOVA) was used to determine differences in final satisfaction scores, according to baseline characteristics, while controlling for the number of sessions completed by the participant.

Program effectiveness was examined using 2 methods. First, changes in anxiety symptom (CAS-8) scores were examined. Paired-samples 2-tailed *t* tests were used to determine changes in anxiety symptoms from baseline to the user’s final recorded anxiety rating, irrespective of the number of completed sessions. That is, if the user completed all 10 sessions, their final anxiety score would be at session 10. If the user completed only 4 sessions, their final anxiety score would be at session 4. In this way, the change in anxiety captures the change made by the person according to the amount of treatment they received. This analysis was conducted for all users including those with and without elevated anxiety when registering.

Given that few participants provided data at all assessment points, we also analyzed the full sample through mixed modeling. The sample was split by elevated or nonelevated baseline anxiety, using time (baseline CAS-8 and final CAS-8 score) as a fixed effect and a random intercept for participants, to confirm the findings of the completer analysis for both primary and secondary analyses, especially among those with elevated anxiety. A sensitivity analysis repeating the mixed models with those participants who completed all 10 sessions of the program was performed to assess the robustness of the analysis to missing data. Furthermore, to investigate changes in anxiety scores at specific points in the program (baseline to session 4, baseline to session 7, and baseline to session 10), separate repeated measures ANOVA tests were conducted for those who had elevated anxiety at program registration and had completed the sessions (eg, sessions 1‐3) and CAS-8 measures up to that point.

Second, the proportion of users making clinically meaningful change on the CAS-8 from their first to last session was calculated for participants shifting from elevated or clinical categories at baseline to nonelevated anxiety or elevated anxiety at their last session. The degree of clinically meaningful change was also examined for users with “normal” anxiety classification at baseline; specifically, to identify the proportion of participants who showed meaningful deterioration into an elevated or clinical category, from their first to last session on the CAS-8 measure who shifted.

## Results

### Implementation and Effectiveness Outcomes

#### Adoption

From 2015 to 2024, a total of 53,726 unique users registered for the BRAVE Self-Help program, including 28,700 child users (mean 9.36, SD 1.40 years) and 25,026 adolescent users (mean 14.15, SD 1.62 years). In terms of total registered users, 27,210 [Bibr R20](94.8%) child users and 24,511 (97.9%) adolescent users completed the baseline assessment, and 22,473 (78.3%) child users and 17,296 (70.6%) adolescent users commenced treatment. Figure 2 maps service use across the 10 years, highlighting notable increases in annual service utilization, particularly between 2019 and 2022. This trend is followed by a gradual decline in 2023, returning to levels comparable to those observed before 2019. Of note, these trends appear to align with Australian community- and nationwide crises experienced in the last 10 years, including the prolonged bushfires in 2019, extensive lockdowns in 2020 and 2021, and multiple flooding incidents across Australian states in 2022.

**Figure 2.**
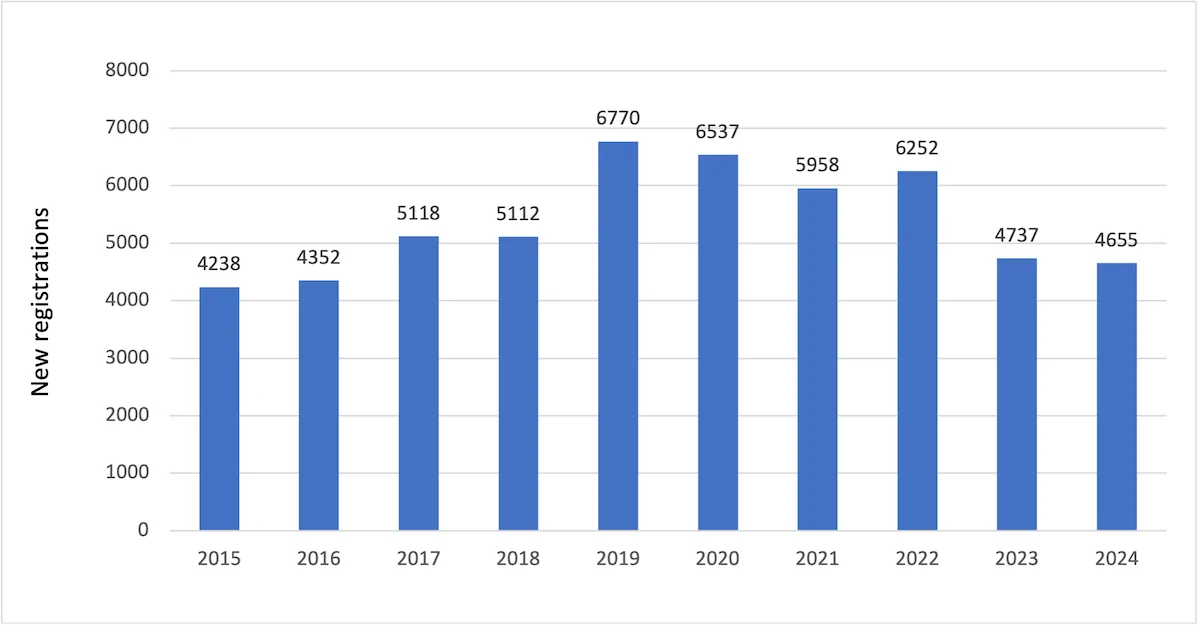
Annual new registrations for BRAVE Self-Help from January 1, 2015, to December 31, 2024.

Detailed monthly and yearly trends are presented in Figure 3 and show a clear pattern of increased user registrations in response to school term commencement and key community- and nationwide events. For example, Figure 3 shows that registrations for BRAVE Self-Help consistently decreased during school holiday periods and increased sharply following the return to school. Further, there was an obvious spike in user registrations during April 2020, coinciding with the World Health Organization (WHO) confirmation of the COVID-19 pandemic and initiation of national lockdowns. The number of monthly registrations during the active COVID-19 lockdown period (April) exceeded 800, in contrast to the usual ≥600 observed in preceding years.

A negative binomial regression with a log link modeled monthly registration counts over the 10-year period (n=120 months), with the dispersion parameter estimated by maximum likelihood (k=0.036, 95% CI 0.028-0.047). Binary indicators for major weather events and school holidays, a pulse indicator for April 2020, month (entered as a factor to capture seasonality), and year were included as predictors. The model fitted well (deviance/df =1.17) and improved on the intercept-only model (*χ*^2^_LR,15_=148.84; *P*<.001). Registrations were 15% lower in school holiday months (incidence rate ratio [IRR] 0.85, 95% CI 0.74-0.98; *P*=.03), approximately twice as high in April 2020 (IRR 2.16, 95% CI 1.45-3.23; *P*<.001), and increased by 2% per year (IRR 1.02, 95% CI 1.01-1.03; *P*=.004). Months containing major weather events showed a 9% higher registration rate that did not reach significance (IRR 1.09, 95% CI 0.99-1.20; *P*=.09). Because the Pearson residuals showed first-order autocorrelation (*r*=0.51), the model was refitted as a generalized estimating equation with an AR(1) working correlation structure and robust standard errors [29]. Conclusions for school holidays, the April 2020 pulse, and the yearly and seasonal trends were unchanged, while the weather event association reached significance in this model (IRR 1.14, 95% CI 1.03-1.26; *P*=.01). Given that 94 of the 120 months contained a major weather event, limiting the available contrast for this predictor, and that the sensitivity model relied on robust standard errors estimated from 10 year-level clusters, the weather association should be interpreted as tentative evidence of a small positive effect.

**Figure 3.**
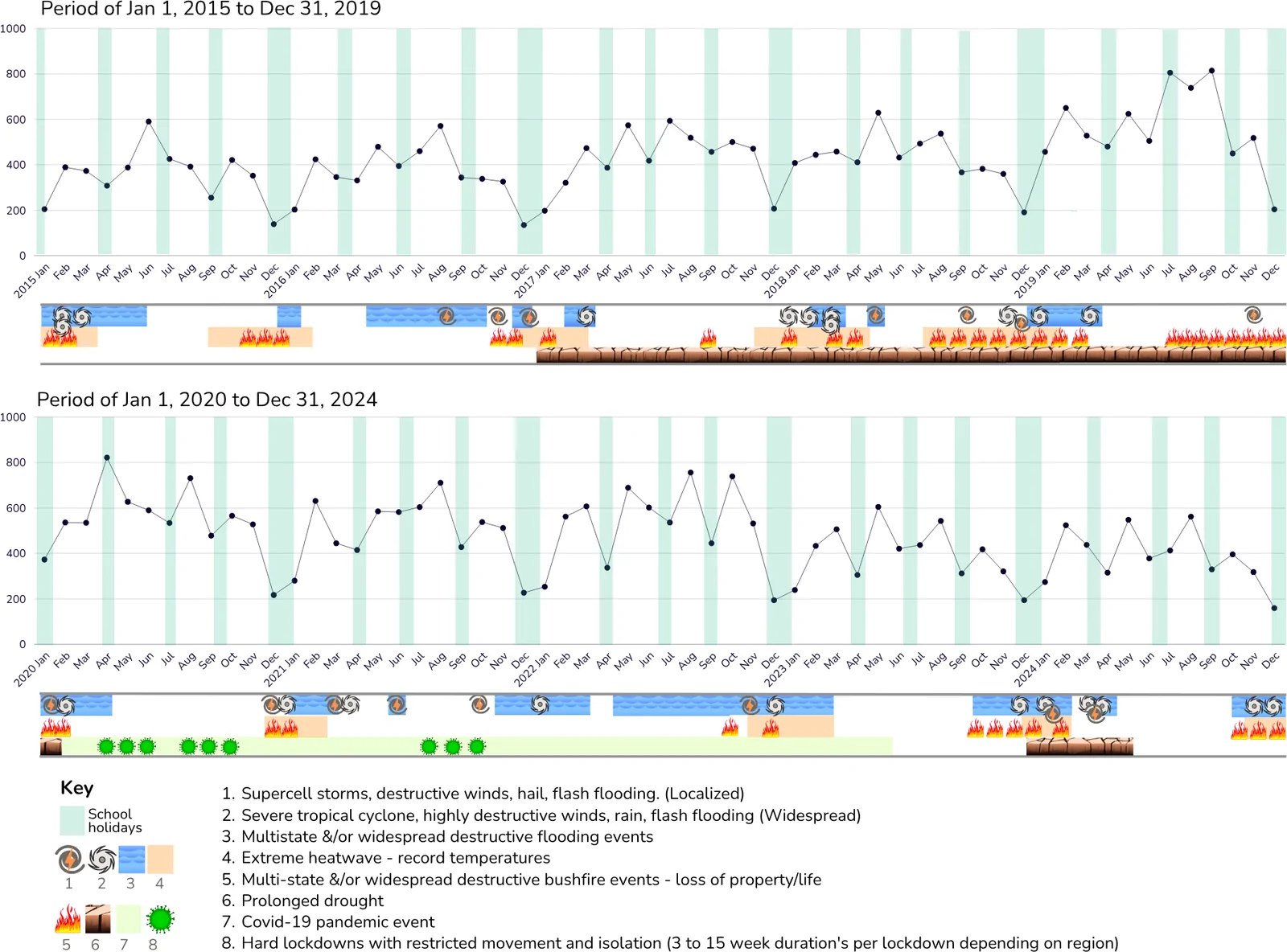
A breakdown of monthly and yearly trends of user registration in response to key community- and nationwide events and school terms.

#### Penetration

The penetration of BRAVE Self-Help is evidenced through its ability to reach young people who differed greatly in terms of age, gender, geographical location, and baseline severity. Further, young people were referred into the program through diverse means, highlighting that the program penetrated community, health, and education settings. Table 2 provides an overview of user registrations, demographic background, referral sources, and clinical profiles.

**Table 2.** Demographic and clinical profile of child and adolescent users from January 1, 2015, to December 31, 2024^a^.

Characteristics		All participants	Children	Adolescents
Registered, n (%)		53,726	28,700 (52.8)	25,026 (47.2)
Baseline completed, n (%)		51,721	27,210 (52.6)	24,511 (47.4)
Age in years, mean (SD)		11.59 (2.83)	9.36 (1.40)	14.15 (1.62)
Gender, n (%)				
Female		31,542 (60.8)	15,099 (55.4)	16,443 (66.9)
Male		19,228 (37.1)	12,050 (44.2)	7178 (29.2)
Other		1089 (2.1)	124 (0.5)	965 (3.9)
Indigenous status, n (%)				
Indigenous		2192 (4.1)	1144 (4)	1048 (4.2)
Not Indigenous		31,553 (58.7)	17,593 (61.3)	13,960 (55.8)
Missing		19,981 (37.2)	9963 (34.7)	10,018 *(*40)
CAS-8^b^, mean (SD)		11.34 (4.90)	10.61 (4.45)	12.16 (5.24)
Presenting anxiety severity, n (%)				
Elevated anxiety		27,076 (52.5)	12,699 (46.8)	14,377 (58.7)
No elevated anxiety		24,543 (47.5)	14,435 (53.2)	10,108 (41.3)
Remoteness, n (%)				
Urban		31,369 (58.4)	16,623 (57.9)	14,746 (58.9)
Regional		14,865 (27.7)	8153 (28.4)	6712 (26.8)
Missing		7492 (13.9)	3924 (13.7)	3568 (14.3)
Referral source, n (%)				
School-based professional		22,992 (45.3)	10,909 (40.6)	12,083 (50.6)
Health professional		11,737 (23.1)	6862 (25.5)	4875 (20.4)
Parent or family member		7401 (14.6)	4279 (15.9)	3122 (13.1)
Beyondblue		1496 (2.9)	608 (2.3)	888 (3.7)
Internet search		3371 (6.6)	2006 (7.5)	1365 (5.7)
Other		3758 (7.4)	2210 (8.2)	1548 (6.5)
Health professional referral, n (%)				
General practitioner		2712 (23)	1590 (23.1)	1122 (22.9)
Psychologist		5844 (49.6)	3522 (51.2)	2322 (47.3)
Social worker		785 (6.7)	380 (5.5)	405 (8.3)
Occupational therapist		628 (5.3)	372 (5.4)	256 (5.2)
Other health professional		1818 (15.5)	1018 (14.8)	800 (16.4)

a Available data are shown in each cell. Not all participants provided an answer on remoteness and Indigenous status. Valid percentages are shown.

b CAS-8: Children’s Anxiety Scale 8-item.

#### Program Use by User Characteristics

A breakdown of overall trends in program use and presenting anxiety severity is provided by user type, gender, and remoteness (Figures S1-S6 in Multimedia Appendix 2). For example, child users and users of other gender showed consistent increases in annual registrations throughout the first 8 years of service. The average presenting anxiety severity of all users remained relatively stable over the project period, although it was notably higher for adolescents compared to children, and for other and female gender compared to male gender.

#### Fidelity and Feasibility

The average number of sessions completed by all users (children and adolescents) varied over the 10-year period (Figure 4), with the average number of sessions completed being 2.67 (SD 2.99) sessions. The average number of sessions completed increased slightly over the project period. Overall, users with elevated anxiety (n=27,076) completed an average of 2.57 (SD 2.89) sessions, while users with no elevated anxiety (n=24,543) completed an average of 2.93 (SD 3.10) sessions. One quarter of all users (13,957/53,726, 26%) completed zero sessions, 36.7% (19,743/53,726) completed 1 or 2 sessions, and 37.3% (20,026/53,726) completed 3 or more sessions (see Figure 5 for a breakdown of all sessions). For participants who started the program (ie, completed 1 or more sessions; n=39,769), the average number of sessions completed by all users was 3.60 (SD 2.95).

**Figure 4.**
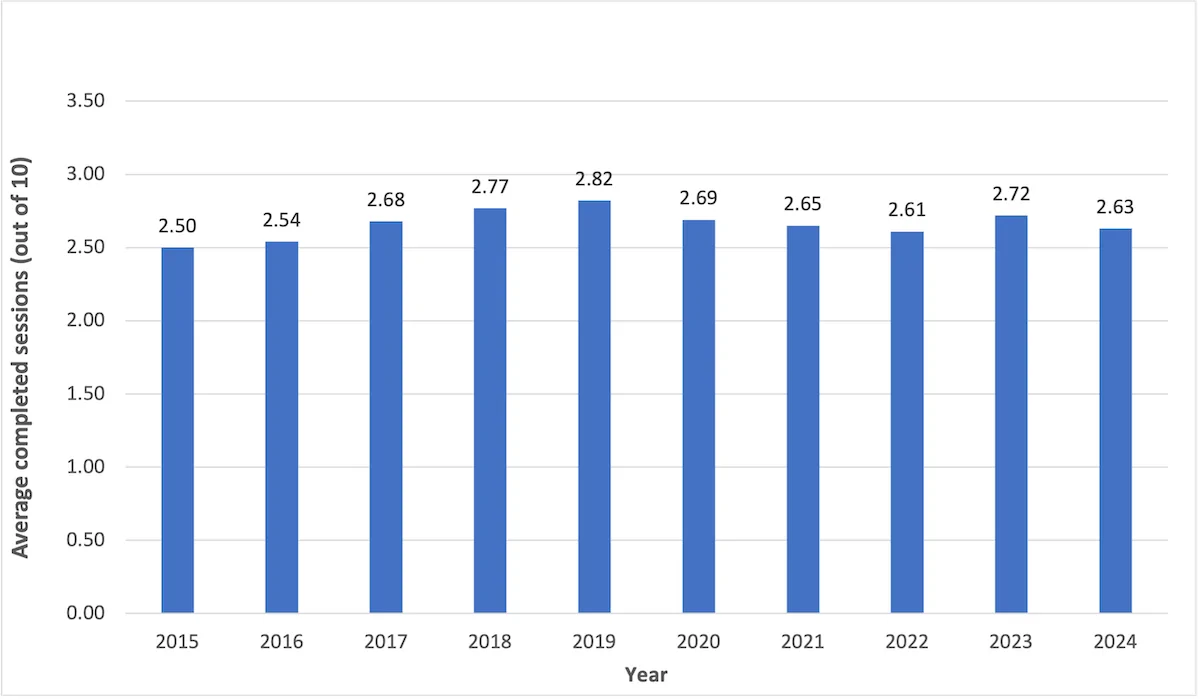
Trends in number of sessions completed per year by all users.

**Figure 5.**
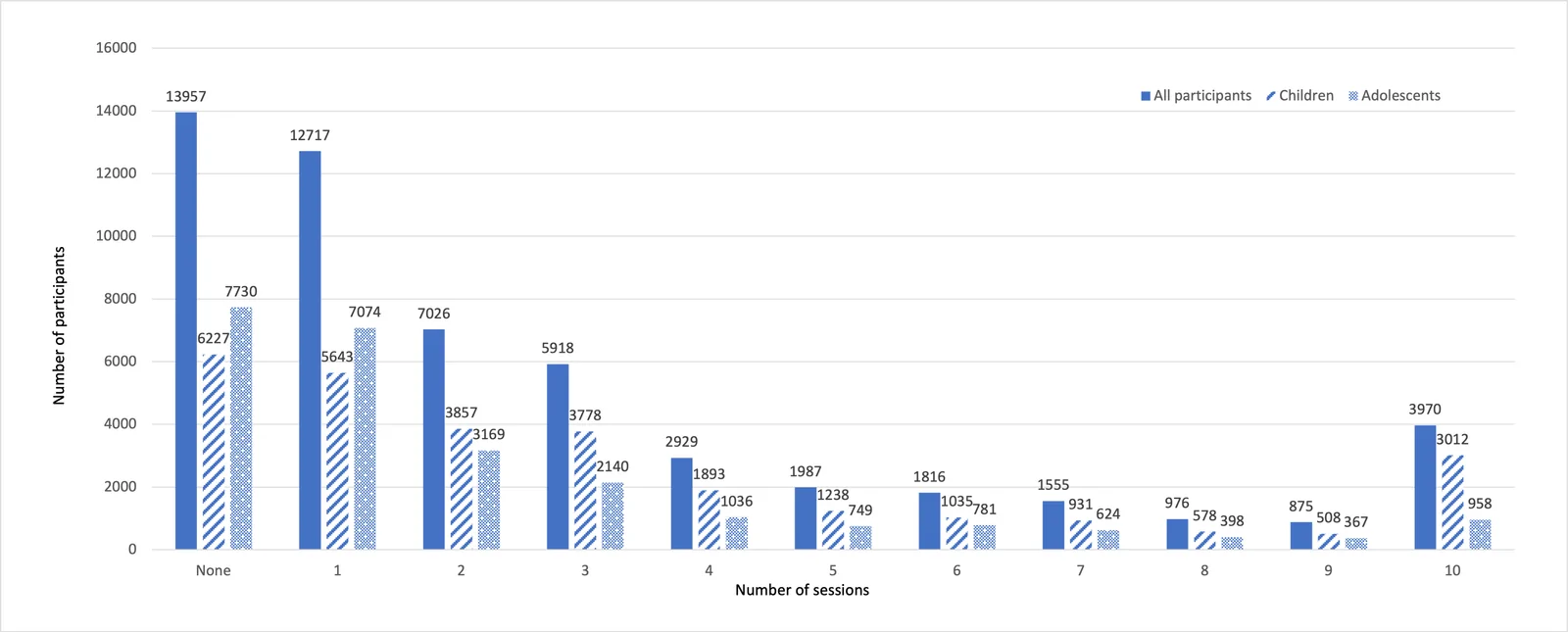
Session completion breakdown for child and adolescent users from January 1, 2015, to December 31, 2024.

#### Attrition and Selection Bias

Chi-square analyses were conducted to compare baseline characteristics between program completers (10 sessions) and noncompleters (<10 sessions). As shown in Table S1 in Multimedia Appendix 2, there were significant associations between completer status and baseline anxiety, referral source, gender, remoteness, and age. Higher completion rates were observed for those with nonelevated baseline anxiety, those referred by school-based professionals, younger (child program), boys, and those living in a regional area. Comparisons between users who did not start the program (completed 0 sessions) and those who completed 1 or more sessions were also performed. As demonstrated in Table S2 in Multimedia Appendix 2, program commencement was significantly associated with all baseline characteristics. A greater proportion of participants who were not Indigenous, did not have elevated anxiety at baseline, were referred by school-based professionals or a friend or family member, were younger (child program), were female, and were living in a regional area completed 1 or more sessions.

Similarly, Table S3 (Multimedia Appendix 2) shows significant associations between engagement level and all baseline characteristics. Higher engagement (4‐10 sessions completed) was associated with nonelevated anxiety, referral by school-based professionals, male gender, younger age (child program), and regional location. Nonengagement (0 sessions completed) was associated with elevated anxiety, Indigenous status and adolescence (adolescent program). Moderate engagement (1‐3 sessions completed) was associated with higher proportions of girls, referrals from health professionals and friends or family members, and urban location. Although engagement level was significantly associated with baseline characteristics, effect sizes were small; the greatest associations were observed for age (*V*=.152) and referral source (*V*=.109), suggesting that these factors were most strongly related to program engagement. Collectively, these findings suggest that, while participants with different levels of engagement did differ on some baseline characteristics, the magnitude of most associations was small.

#### Reasons for Nonadherence

To add further context to program completion rates, a feedback survey completed by a subsample of users (n=413) during a 3-month period in 2015 provided data regarding reasons for nonadherence early in program delivery (Multimedia Appendix 3). The reasons reported by participants for ceasing participation in the program are detailed in Table 3. The most common reason cited by both children and adolescents was that reduction in symptoms during the program may cause participants to determine that they do not need to continue with the program.

**Table 3.** Self-reported reasons for nonadherence in subsample (n=413) of participants.

Reason for nonadherence	Children (n=173), n (%)	Adolescents (n=113), n (%)
Reduction in anxiety symptoms	88 (50.87)	44 (38.94)
Boredom or lack of motivation	20 (11.56)	22 (19.47)
Feeling overwhelmed	4 (2.31)	4 (3.54)
Increased anxiety	1 (0.58)	1 (0.88)
Finding the program too difficult	1 (0.58)	2 (1.77)
Time constraints	18 (10.40)	8 (7.08)
Length of sessions/program	6 (3.47)	10 (8.85)
Feeling the program wasn’t helping	14 (8.09)	12 (10.62)
Technical issues	4 (2.31)	0 (0)
Homework	2 (1.16)	0 (0)
Seeking other help	0 (0)	4 (3.54)
Embarrassment	0 (0)	2 (1.77)
Other/unclassifiable responses^a^	15 (8.67)	4 (3.54)

a Percentages are based on valid responses. Reasons for nonadherence were reported by 173/241 children and 113/172 adolescents.

#### Appropriateness and Acceptability

Program satisfaction was relatively stable over the project period (Figure 6). Users reported moderate to high total satisfaction with the program. Figures 7–9 present mean satisfaction scores for individual acceptability items for users who completed 3, 6, or 9 sessions, including whether users would refer a friend to the program, helpfulness of the program, happiness with the program, and overall satisfaction with the program. Children generally provided higher ratings of acceptability and helpfulness than adolescents, although all ratings were in the moderate to high range. Approximately 68.3% (7200/10,537) of children and 53.1% (3073/5784) of adolescents reported that the overall program was good or very good, and 56.6% (6044/10,673) of children and 52% (3040/5849) of adolescents answered “yes” or “definitely yes” when asked if they would tell a friend about the program if that friend was worried or anxious.

**Figure 6.**
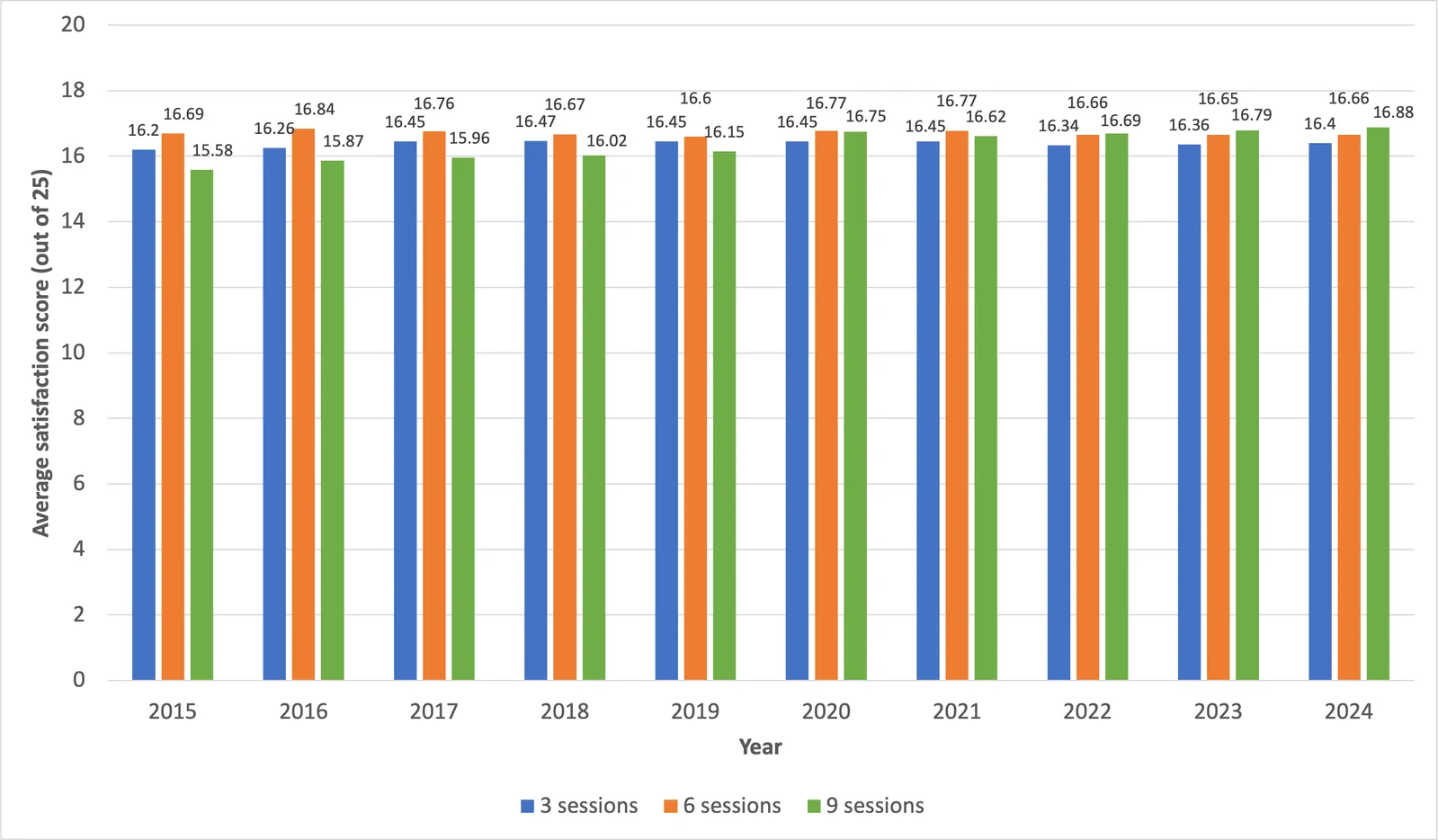
Overall satisfaction with the program from January 1, 2015, to December 31, 2024, for users who completed 3, 6, or 9 sessions in total.

**Figure 7.**
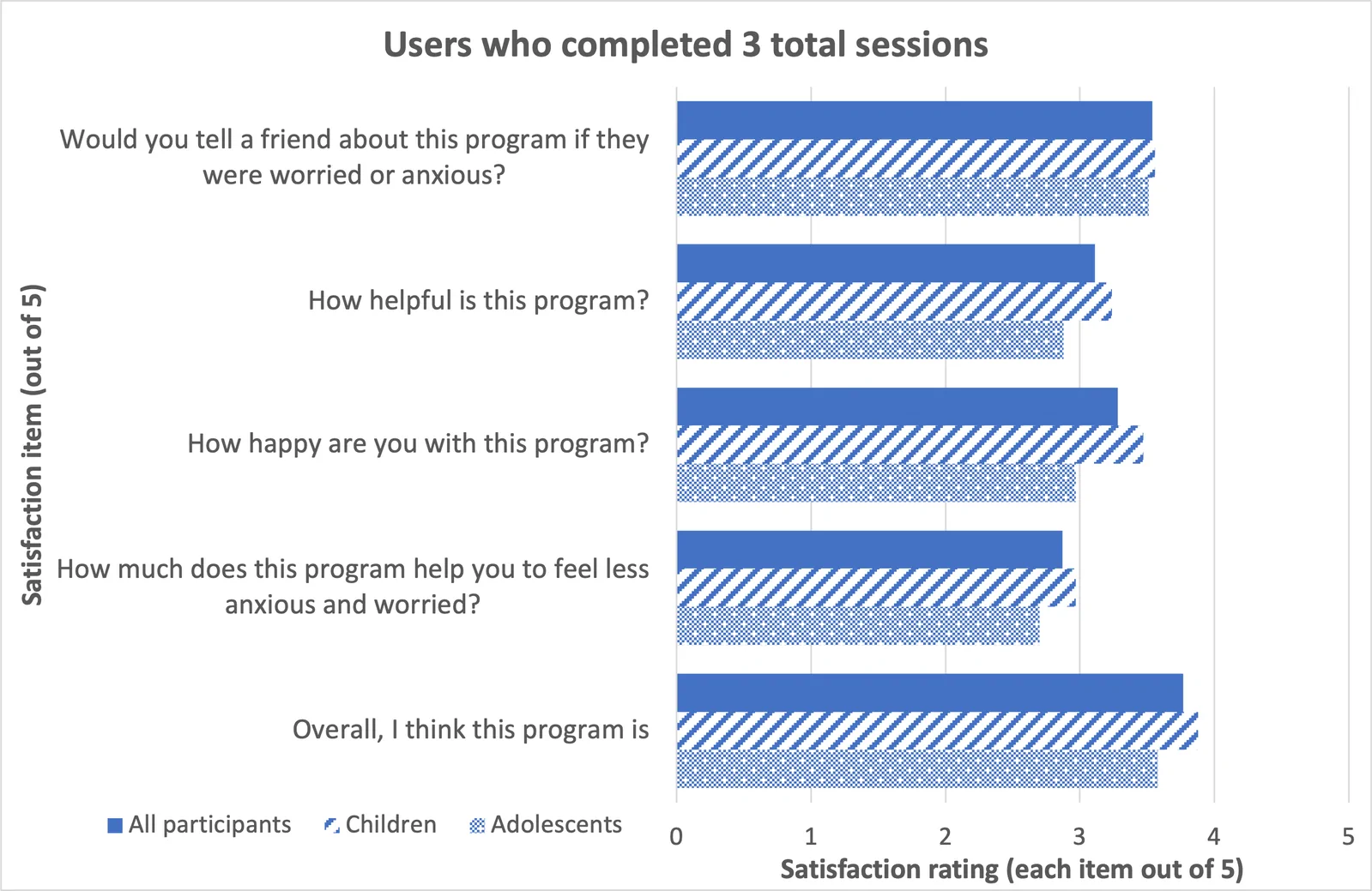
Mean satisfaction ratings for individual satisfaction items for users who completed 3 total sessions from January 1, 2015, to December 31, 2024.

**Figure 8.**
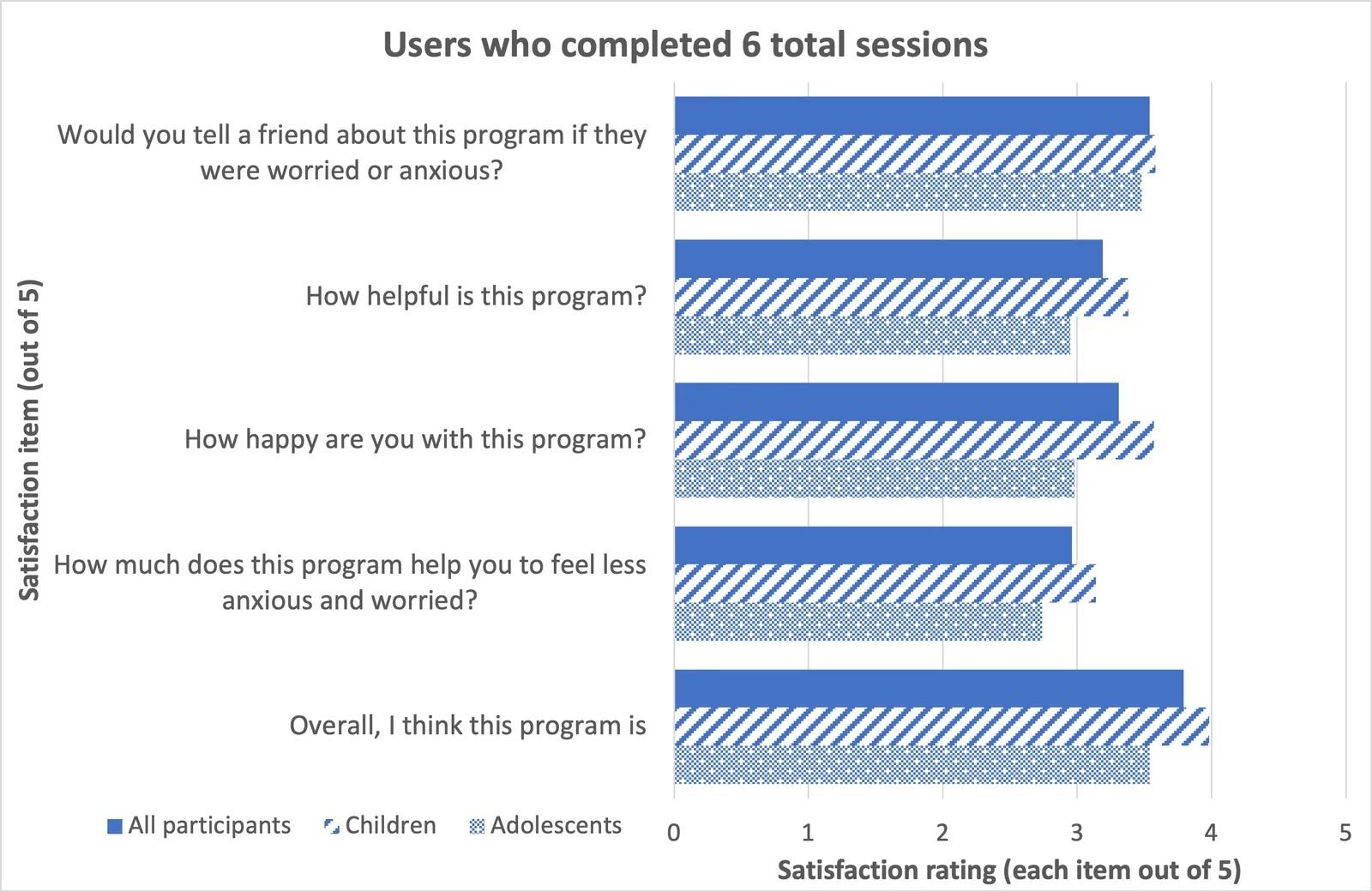
Mean satisfaction ratings for individual satisfaction items for users who completed 6 total sessions from January 1, 2015, to December 31, 2024.

A one-way ANCOVA was used to determine differences in final satisfaction scores, according to baseline characteristics, while controlling for the number of sessions completed by the participant. The overall model was significant, accounting for 5.5% of the variance in satisfaction scores (*F*_13,9251_=41.78, *R*^2^=.055; *P*<.001). Referral source (*F*_5,9251_=6.75, ηp^2^=.004; *P*<.001), gender (*F*_2,9251_=17.58, ηp^2^=.004; *P*<.001), program type (*F*_1,9251_=252.51, ηp^2^=.027; *P*<.001), and sessions completed (*F*_1,9251_=165.07, ηp^2^=.018; *P*<.001) all significantly contributed to the model. Post hoc comparisons showed that participants referred by school-based professionals (Δ*M*=16.18) showed lower satisfaction than participants referred by health professionals (Δ*M*=16.64) and participants who found the program via an internet search (Δ*M*=17.42); child program participants reported greater satisfaction (Δ*M*=17.64) than adolescent participants (Δ*M*=15.78), and girls (Δ*M*=17.34) reported greater satisfaction than boys (Δ*M* 16.76).

Results from the feedback survey completed by the subsample of children and adolescents in 2015 provided further data related to acceptability. Of the 241 children and 172 adolescents who completed the feedback survey, most children (193/241, 80%) and adolescents (136/172, 79%) reported that the program had met their expectations. In terms of program components they found most helpful, 47.7% of children (115/241) and 37.6% of adolescents (65/172) reported that the program was helpful in teaching them strategies and reducing their anxiety.

**Figure 9.**
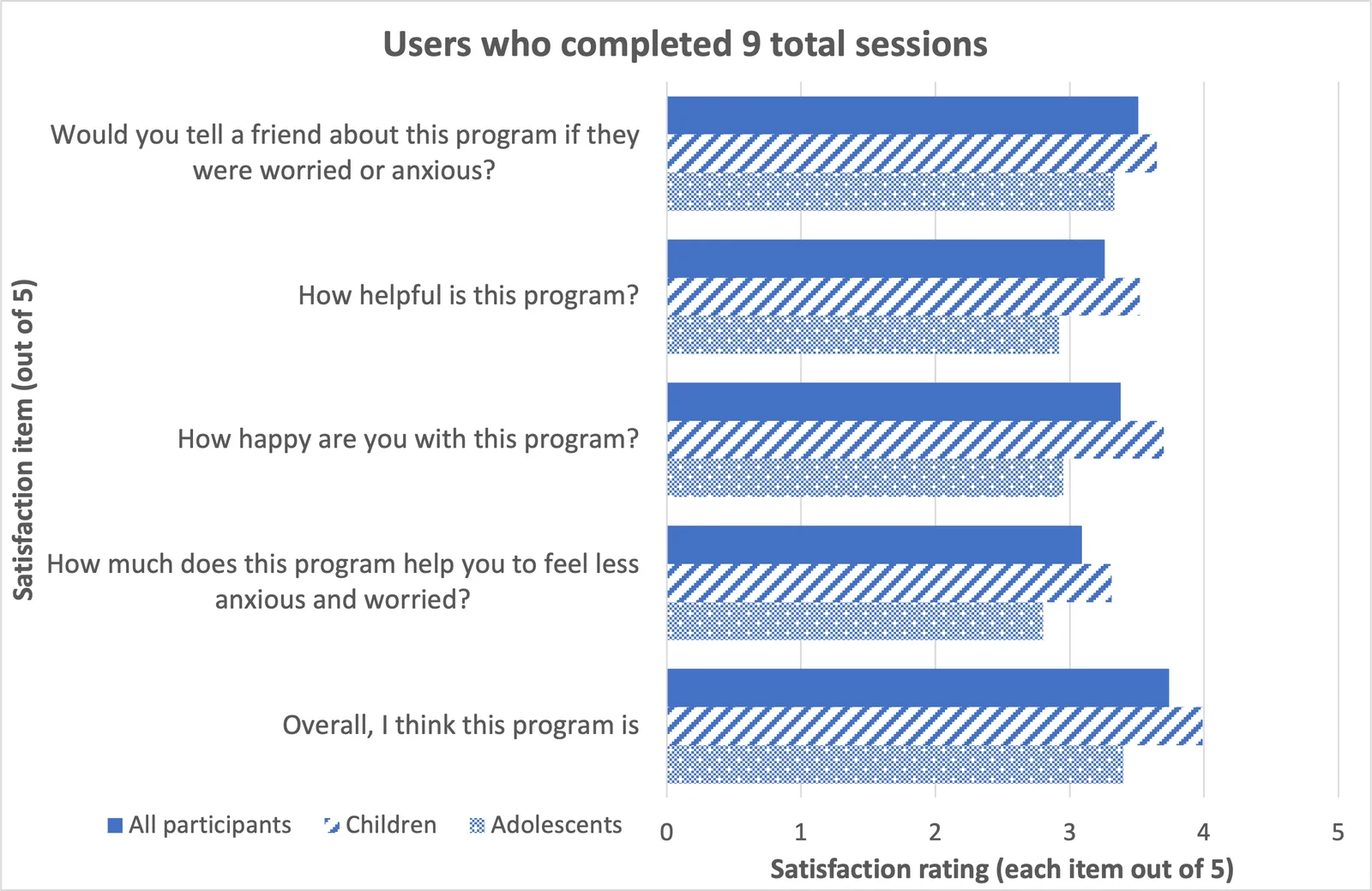
Mean satisfaction ratings for individual satisfaction items for users who completed 9 total sessions from January 1, 2015, to December 31, 2024.

#### Effectiveness

##### Changes in Anxiety Severity

A 2-tailed paired-samples *t* test demonstrated a significant reduction in CAS-8 scores from baseline to final CAS-8 scores (irrespective of the number of sessions completed), with a medium to large effect size for users with elevated anxiety, Cohen *d*=0.54 (Figure 10). Significant improvements were evident for all users (Cohen *d*=0.33), although (as would be expected) the magnitude of the effect was very small for users without elevated anxiety at baseline, Cohen *d*=0.08 (Table S1 in Multimedia Appendix 2).

**Figure 10.**
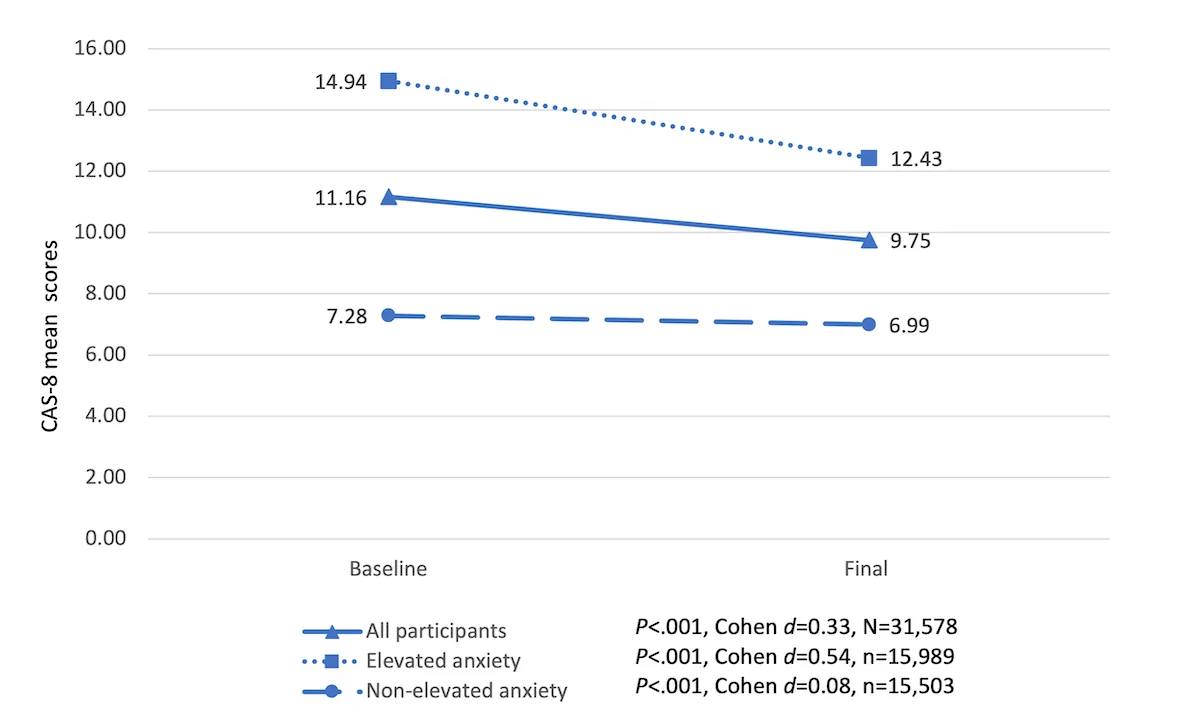
Effectiveness of the program from baseline to final CAS-8 score. CAS-8: Children’s Anxiety Scale 8-item.

In addition, results of the mixed modeling analyses confirmed findings from the completer sample and demonstrated significant anxiety improvement over time (Table S2 in Multimedia Appendix 2). The models contained a random intercept for subject ID and a fixed effect for time (baseline CAS-8 and final CAS-8). The restricted maximum likelihood (REML) method was used, and data were assumed to be missing at random (MAR). For those with elevated anxiety at baseline, there was a significant effect of time on CAS-8 scores (*F*_1, 22,613.47_=5966.53; *P*<.001). A significant decrease was demonstrated from baseline to final CAS-8 score (*b*=–2.57, SE 0.03; *t*_22,613.46_=–77.24; 95% CI –2.64 to –2.51; *P*<.001). For participants with nonelevated baseline anxiety, there was a significant effect of time (*F*_1, 21,074.64_=112.18; *P*<.001). The reduction in CAS-8 scores from baseline to final CAS-8 score was significant, although smaller, for these participants (*b*=–0.28, SE 0.03; *t*_21,074.64_=–10.59; 95% CI –0.33 to –0.23; *P*<.001).

The mixed models were rerun using participants who completed all 10 sessions of the program. In this sensitivity analysis, reductions in CAS-8 scores were observed among both elevated and nonelevated participants. For participants with elevated baseline anxiety, there was a significant effect of time on CAS-8 scores (*F*_1, 1786_=1339.53, *P*<.001). CAS-8 scores decreased from 14.78 (SE 0.08, 95% CI 14.62-14.93) at baseline to 9.55 (SE 0.14, 95% CI 9.27-9.83) at session 10. The estimated mean reduction was 5.23 points (SE 0.14, 95% CI 4.95-5.51). Among those participants with nonelevated baseline anxiety, there was also a significant effect of time on CAS-8 scores (*F*_1, 2131_=161.22; *P*<.001). CAS-8 scores decreased from 6.83 (SE 0.06, 95% CI 6.72-6.95) at baseline to 5.62 (SE 0.10, 95% CI 5.43-5.81) at session 10. The estimated mean reduction was 1.22 points (SE 0.10, 95% CI 1.03-1.41; *P*<.001). These findings were consistent with the primary mixed-model analyses, suggesting that the observed improvements in anxiety symptoms were robust to missing data.

When examining changes in anxiety from baseline to session 4, baseline to session 7, and baseline to session 10 for those with elevated anxiety who had completed sessions (Figure S7 in Multimedia Appendix 2), significant reductions were noted, with large effect sizes (approximately *η*^2^=.25, *η*^2^=.27, and *η*^2^=.28, respectively). Users who completed 6 or more sessions demonstrated a 4-point improvement in CAS-8 scores, and effects increased with more sessions completed.

To examine whether baseline anxiety scores predicted final anxiety scores, while controlling for the number of sessions, a multiple linear regression analysis was conducted. The overall model was statistically significant (*F*_2, 31,575_=13,193.13; *P*<.001), explaining 45.5% of the variance in final anxiety scores (*R*^2^=.455, adjusted *R*^2^=.455). Baseline anxiety scores were a significant predictor of final anxiety scores (*b*=0.71, SE 0.005, *β*=.636, *t*=152.63, 95% CI 0.700-0.718; *P*<.001). The number of sessions completed was also a significant predictor of final anxiety scores (*b*=–0.33, SE 0.008, *β*=–.181, *t*=−43.49, 95% CI –0.343 to –0.314; *P*<.001), indicating that completing more sessions was associated with a lower final anxiety score after controlling for baseline anxiety.

##### Clinically Meaningful Change

Clinically meaningful improvement was considered evident if participants moved from a clinical level of anxiety at baseline to either elevated or nonelevated anxiety at their final session, or from an elevated level at baseline to a nonelevated level at their final session. Overall, 52.75% (7779/14,746) of participants demonstrated clinically meaningful change. Table S3 in Multimedia Appendix 2 shows the proportion of users who exhibited clinically meaningful change by baseline anxiety level and age. The proportion of children who crossed the elevated threshold was significantly greater than for adolescents (*χ*^2^_6978_=127.7, *V*=.135; *P*<.001). Among children who presented with elevated anxiety, 63.9% (2581/4036) demonstrated recovery and crossed to the nonelevated range at their final session. Over half of adolescents with elevated anxiety at baseline (1484/2942, 50.4%) shifted to nonelevated at their final session.

Among users with clinical levels of anxiety, 56.3% (2162/3842) of children and 39.5% (1552/3926) of adolescents crossed the threshold from clinical level to either the elevated or nonelevated range. There were significantly more adolescents than children who remained clinical, and the proportion of adolescents who demonstrated recovery into the nonelevated range was significantly less (*χ*^2^_7,768_=333.3, *V*=.207; *P*<.001; Table S4 in Multimedia Appendix 2). There was no difference between the proportion of children (885/3842, 23%) and adolescents (916/3926, 23.3%) who demonstrated a meaningful response to treatment and reduced from the clinical to the elevated range. Additionally, 33.2% (1277/3842) of children and 16.2% (636/3926) of adolescents reported CAS-8 scores indicative of recovery and reduced from the clinical to nonelevated range. Figure 11 shows the proportions of clinically meaningful change among children and adolescents in the elevated or clinical ranges at baseline.

The proportion of participants who presented with nonelevated levels of anxiety at baseline and who deteriorated into elevated or clinical levels of anxiety was 13.59% (1351/9942) of children and 12.82% (713/5561) of adolescents. There was no significant difference overall in the proportions of children and adolescents who remained nonelevated or who deteriorated to elevated or clinical anxiety levels at their final CAS-8 score (*χ*^2^_15,503_=4.7, *V*=.017; *P*=.10; Table S5 in Multimedia Appendix 2).

**Figure 11.**
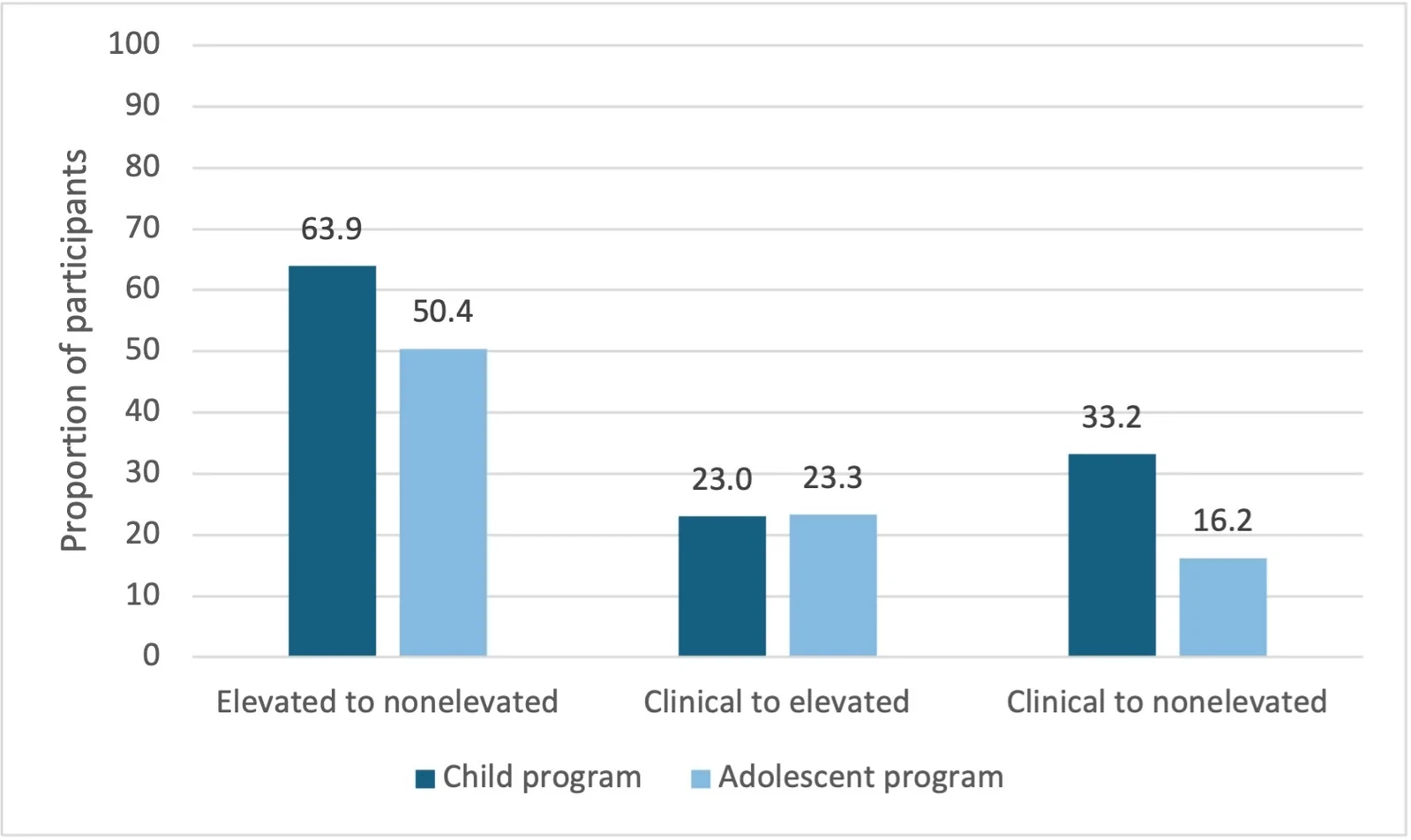
Proportions of meaningful improvement for children and adolescents with elevated or clinical anxiety levels at baseline.

## Discussion

### Main Findings

This study summarized the implementation and effectiveness outcomes of the BRAVE Self-Help program, reaching more than 50,000 users over 10 years of delivery. Despite implementation evaluation methods not being prominent over 10 years ago when this program was first implemented, this study offers a retrospective examination of implementation using available data. In doing so, it acknowledges that some information required to assess implementation according to contemporary standards was not collected. Using an open effectiveness-implementation trial design with a large Australian child and adolescent sample, this study represents one of the first demonstrations, within pediatric mental health, of how a publicly available DMHP for child and adolescent anxiety can be implemented nationally using a strategic approach grounded in best-practice implementation frameworks. This platform, delivering an evidence-based therapeutic program, is distinct from other national telephone or digital crisis support services (eg, Lifeline and Kids Helpline), which offer in-the-moment support for distress, rather than complete online CBT interventions. The findings show that BRAVE Self-Help is a scalable, responsive and effective approach for improving anxiety symptoms among large numbers of young people, and has been, and continues to be, able to provide support in times of community- and nationwide stressors.

### Implementation Context and Strategies

The findings of this study highlight the importance of involving education and health professionals in any implementation and dissemination efforts for programs involving child and adolescent mental health. Two-thirds of participants were referred into the program via school (eg, guidance counselors or school psychologists) or health professionals, with the latter most likely to be referred by Psychologists. The tendency for people to find the program via professional rather than self-referral highlights the potential for DMHPs to provide service support to mental health professionals, potentially support waitlist reduction, and assist in directing evidence-based support to young people who might otherwise not be able or willing to receive it. Successful implementation of DMHPs for young people in the community will require implementation strategies directly integrating school and health professionals. Although this study did not explicitly assess the impact of its implementation strategies (Multimedia Appendix 1) on implementation outcomes, the strategies were well aligned with recommended effective implementation strategies [18] and could provide examples for future digital models of service delivery, policymakers, and relevant stakeholders.

### Sustained Program Use With Periods of Growth

A key observation was the notable increases in program use aligning with significant community- and nationwide stressors, in particular, during the COVID-19 pandemic and significant weather events. This underscores two critical points: (1) an escalating demand for accessible digital services addressing youth mental health needs, and (2) the ability of the BRAVE Self-Help program to respond to local, national, and global events. This study’s findings are consistent with broader evidence indicating a rise in youth mental health problems that may have subsequently led to a trend of increasing service use, particularly in the context of nationwide stressors [30]. The overall increasing program use trend observed here is also consistent with evidence from other national DMHP platforms in Australia [4,31]. The findings underscore the growing relevance of programs such as the BRAVE Self-Help DMHP in meeting the needs of young Australians, including the potential to address the evolving mental health landscape aggravated by societal challenges.

### Ability to Reach Diverse Groups of Young People

The results of this study demonstrate that BRAVE Self-Help was able to reach a demographically and clinically diverse cohort of young people. The program reached young people across vast geographic areas, including substantial proportions of young people from regional and rural areas who typically only have limited access to evidence-based mental health services. The program was also able to reach young people with mild, moderate, and severe levels of anxiety, with over half experiencing levels of anxiety that were causing problems for the young person (eg, indicative of an anxiety condition). Though overall there were more female than male users, the program was able to reach male adolescents (30% of adolescent users) and male children (44% of child users). Consistent with school-based research [24], girls presented with higher anxiety symptom severity than boys, and adolescents generally reported higher anxiety severity than children. Importantly, during implementation, the program was able to reach young people identifying as nonbinary or other gender (3.9% of adolescent users), who also presented with notably higher levels of anxiety severity than boys and girls. The proportion of users identifying as other genders increased throughout the extended pandemic (2020‐2022), coinciding with higher levels of anxiety, although the sample size is small. There is a need for further research to explore the suitability of DMHPs for priority populations.

It is worth noting that although adolescent users demonstrated higher levels of anxiety than children over 10 years of service delivery, especially during the extended pandemic, their use of the program reduced in the last 4 years, while child use continued to grow. A potential explanation for reduced use of DMHPs by this cohort could be attributed to prolonged digital fatigue and social isolation through the extended pandemic period, which may have been particularly pronounced for adolescents who are in a developmental stage characterized by an increased need for peer interaction [32]. Moreover, child users may be more likely to have parental assistance in seeking this model of care and adhering to the BRAVE Self-Help program than adolescent users. Therefore, this study offers insights into the specific groups of young people prone to heightened anxiety levels and those more inclined to demonstrate increased program use. These findings can inform future tailored service delivery strategies for disaster preparedness efforts among potentially at-risk groups.

### Acceptable and Effective Options for Many Young People

The BRAVE Self-Help program consistently demonstrated acceptability and effectiveness in anxiety symptom reduction for children and adolescents. It was interesting to note that participants referred via school-based professionals had slightly lower satisfaction than those referred by health professionals, or those who self-referred after finding the program online. This may be because schools were using the program as part of curriculum delivery (and therefore may not have been appropriate for some young people) or that they referred young people inappropriately to the program. Although effectiveness was higher for children than adolescents, it is important to note that adolescents also presented to the program with higher anxiety severity, likely entrenched over several years from childhood to adolescence. For example, 63% of children and 50% of adolescents with elevated anxiety at program registration achieved clinically meaningful improvements in their anxiety throughout program participation. This finding is consistent with our previous BRAVE Self-Help effectiveness outcomes, with a comparable medium to large effect size noted for users presenting with elevated anxiety [9]. This study also demonstrates that users with no elevated anxiety showed significant reductions in anxiety following program participation, although this effect was small. Some originally nonanxious participants showed deterioration through the study. It is important to note that BRAVE Self-Help was not developed as a prevention intervention, but rather as an early treatment for existing symptoms of anxiety. Therefore, the sessions and strategies were not targeted at preventing new anxiety development. It is likely that it was difficult for such participants to implement strategies given they did not initially present with anxiety.

Overall, the findings affirm the potential effectiveness of this digital self-help intervention for managing anxiety in young people and further support the role of DMHPs as a viable public health and population-level delivery model. Considering the substantial estimated demand for mental health care among Australian children and adolescents, including those without indication of need (such as those without mental health disorders in the past 12 months or with subthreshold mental health problems) [33], our findings suggest that BRAVE Self-Help has the potential to reach a broader spectrum of young people with varying needs. The platform can offer both preventive and early intervention approaches, leading to effective symptom improvement.

### Brief Program Participation Can Bring About Positive Effects

It is crucial to acknowledge the generally lower engagement rates observed in DMHPs, including BRAVE Self-Help. While average completion rates were modest, findings indicate that clinically meaningful reductions in anxiety can occur even with partial program completion, though it is important to acknowledge that this may reflect natural recovery over time and that users may discontinue the program for many reasons. Within the context of a self-directed public health intervention, early disengagement may reflect difficulties progressing through the self-help program on their own; however, it may also reflect symptom improvement or goal attainment rather than treatment failure. Importantly, treatment effects increased with greater program exposure, with the strongest reduction reported among users who completed 6 or more sessions. Identification of young people who require the full treatment program and those who are most likely to disengage for reasons other than symptom improvement will be important in generating clinical recommendations and triaging young people into appropriate services. Importantly, low program engagement for young people is not unique to DMHPs; the average number of sessions attended by young people at youth-focused headspace in-person clinics in Australia is 4.6 sessions, with 53% of participants attending only 1 to 3 sessions [34]. However, even with low engagement, programs such as BRAVE Self-Help can support many young people in achieving symptom reduction; however, the long-term maintenance of such improvements could not be determined in this study. Understanding engagement challenges is crucial for optimizing digital mental health interventions in the future. Strategies that may enhance user engagement have been identified by users of BRAVE-ONLINE, and include personalization of DMHPs, additional professional and community support, and flexible delivery options [35].

### Study Strengths and Limitations

This study included a very large sample and conducted a comprehensive evaluation of implementation and effectiveness outcomes of this DMHP including reach, penetration, acceptability, fidelity, and effectiveness. The implementation and sustainability strategies used for BRAVE Self-Help can inform the dissemination of other DMHPs, and the study provides a potentially replicable evaluation framework for other DMHPs in the future. Additionally, the effectiveness of the program was examined via a robust standardized tool for child and adolescent anxiety (CAS-8), facilitating comparisons with national and global norms. Limitations of this study include the use of an observational study design with no control, meaning we cannot be certain that improvements in anxiety are solely attributable to the intervention. It is possible that the improvements in anxiety symptoms reflect natural recovery over time, or regression to the mean, though it was clear that greater program engagement was associated with greater symptom reduction.

In terms of program completion, it is important to acknowledge that engagement differed according to anxiety severity, location, age, referral sources, and gender. Though our nonadherence survey provided some qualitative insights into reasons for noncompletion, this data were limited to a 3-month subsample of users collected very early in program delivery and may not be generalizable to later enrollments in the program in a vastly different DMHP landscape. Further work is required to identify the reasons for program noncompletion among different young people now participating in the program and to provide guidance as to who self-help programs are most appropriate for. It is also important to acknowledge that findings derived from the Australian context may not readily generalize to other countries due to potential cultural, contextual, and health care systems variations, as well as the unique circumstances occurring during the COVID-19 pandemic. Nevertheless, this study involved large-scale nationwide engagement of Australian children and adolescents in a self-help DMHP.

### Conclusions

The BRAVE Self-Help program is an accessible, scalable, and effective DMHP that provides a viable public health approach to supporting child and adolescent anxiety. The utility of this program remained stable across periods of cumulative community, national, and global stressors, including the COVID-19 pandemic, with consistent completion, satisfaction, and effectiveness outcomes over 10 years of delivery. Contrary to concerns that digital fatigue [36] may have reduced young people’s engagement with online mental health interventions, findings from this study demonstrate sustained demand, acceptability, and effectiveness of DMHPs such as the BRAVE Self-Help program for anxiety.

## Supplementary material

10.2196/97810 Multimedia Appendix 1 Detailed implementation strategies.

10.2196/97810 Multimedia Appendix 2 Implementation outcomes.

10.2196/97810 Multimedia Appendix 3 Feedback survey with young people and school professionals.

## Abbreviations

ANCOVA: analysis of covariance
CAS-8: Children’s Anxiety Scale 8-item
CBT: cognitive behavioral therapy
DMHP: digital mental health program
iCBT: internet-based cognitive behavioral therapy
IOF: implementation outcomes framework
IRR: incidence rate ratio
MAR: missing at random
REML: restricted maximum likelihood
WHO: World Health Organization
