# Supplementary material for: 10 Years of BRAVE Self-Help Service Delivery for Child and Adolescent Anxiety in Australia: Open Effectiveness-Implementation Trial
Source: J Med Internet Res. 2026 Oct 5;28:e97810. doi: 10.2196/97810 (PMC13638004; doi:10.2196/97810)
Supplement: Supplementary file 1 [file jmir-v28-e97810-s001.doc]

Detailed Implementation Strategies

In this study, BRAVE Self-Help was delivered in a community setting, by the BRAVE Team who were based in universities. Thus, implementation strategies were employed at community, regional and national levels, rather than in specific organizational settings. Implementation strategies were also targeted towards increasing child and adolescent user uptake of the program directly, or via referral into the platform. For the latter, this was not confined to a service, but rather attempted to target any contexts in which referral might be facilitated (eg, family, educational, health service, community). Implementation strategies during the Establishment phase occurred prior to launch and during the soft launch phase (2014) as well as the first two full years after full launch of the program (2015-2016). During the Sustainability phase, the program continued to be disseminated without direct external funding (eg, grants or service payments) to support implementation strategies. Table 2 provides a description of implementation strategies utilized in this project across Establishment and Sustainability phases, as mapped to Powell’s recommended Implementation Strategies.

**Table 2.** Summary of implementation strategies mapped to Powell et al’s [18] effective implementation strategies.

| **Implementation Strategy recommended by Powell** | **Description of strategy** |
| --- | --- |
| ***Establishment Phase (2014-2016)*** | |
| Access new funding | Funding was received from a peak national mental health body (beyondblue) to support the establishment of the open access, BRAVE Self-Help program in Australia. This funding also supported the implementation of the program, including strategies to promote awareness, uptake and usage of the program over the establishment phase of the project (first 3 years). |
| Use advisory boards and workgroups | Governance structures were established to ensure successful program implementation and consisted of an advisory panel and project management group who were responsible for program implementation and included representation from stakeholders with clinical, research, digital and lived experience. The governance structure and responsibilities are provided in Multimedia Appendix 1. |
| Centralize technical assistance  Change record systems  Develop and organize quality monitoring systems | To ensure successful implementation of a nation-wide, open-access, program for anxiety, it was essential to establish technical changes required to facilitate implementation. A detailed description of technical adaptations are presented in Multimedia Appendix 1. Key strategies included:   - Develop and implement technical and program interface changes required to optimize readiness for self-help delivery (eg, additional resources for key treatment techniques). - Enhancement of record and administrative system for recording of program metrics (usage, engagement, feedback) to facilitate quality monitoring - Establish in-built systems for immediate technical support and communication (guided FAQs and problem solving) - Enhancement of record system (user details, demographics) |
| Use mass media  Distribute educational materials | A major focus included building awareness of the program in academic, professional and community settings, together with the understanding that the program was open access and free-of-charge. A promotion and dissemination plan was developed in partnership with the funder (see Multimedia Appendix 1 for examples) and included   - development of age-appropriate promotional resources - social media and internet dissemination - media releases at key points of program (eg, launch, 1-year) - integrating the program weblink into relevant stakeholders and services (eg, national health directories) - academic outreach and promotion - distribution of promotional and educational material (via email and letter) to general practitioners, schools, health practitioners and health clinics located in regional areas |
| Conduct educational meetings  Conduct educational outreach visits  Conduct ongoing training | Increasing awareness of the program and delivering training and educational support to professionals who may refer users into the program was a major implementation strategy. This involved:   - developing a profile at conferences/seminars relating to health, mental health and education fields that were attended by relevant professionals - delivering training guides and seminars to education and health professionals in local communities, across regions and nationally - conducting outreach visits to rural education sites |
| Work with educational institutions  Identify and prepare champions  Identify early adopters  Use train-the-trainer strategies | The education context was a crucial setting through which to reach young people, particularly given the internalizing nature of anxiety that makes recognition of problems difficult without familiarity with the child and their behavior. Strategies included:   - ongoing promotion through KidsMatter and MindMatters programs (national programs supported by beyondblue and integrated in school settings) - school promotion via email flashes and hardcopy distribution of resources (promotional flyers and postcards) and distribution to parents via newsletters - participation in professional training days for school-based professionals. This was targeted at school guidance counsellors/psychologists who were potential champions for the program. Train-the-trainer approaches with education professionals were utilized to enhance awareness across regions and nationally. - raising awareness at local school events - surveys with early adopter stakeholders. Specific outcomes included recognition of the different uses of the program in school contexts (eg, some teachers using in universal contexts) that informed education materials for school professionals (see below). - development of an implementation guide for school-based professionals that was available free of charge either in printed or electronic formats. The guide was adapted from the generic professionals training resources after consultation with early education adopters around alternative ways to use the program in school settings. - results of the feedback surveys for young people and School Professionals can be found in Multimedia Appendix 2 and are also discussed in the Feasibility section below. |
| Audit and provide feedback  Use data experts | - Following launch, a data-informed refinement process was established to ensure that the program and ongoing promotional efforts were optimized throughout the Establishment phase. This process was iteratively conducted and was informed by live program metrics and user feedback obtained through program implementation. Refinement included technical and software upgrades, improved data capturing and exporting, and the development of support resources. Multimedia Appendix 1 summarizes the specific data-informed refinements made. - Data experts examined data and metrics at regular intervals to identify trends and establish appropriate monitoring and analysis approaches. For example, given the unrestricted access to the program, the importance of examining outcomes within specific participant groups who utilized the program for different purposes (eg, prevention, early intervention and treatment) was established. Those who entered the program with elevated anxiety showed much larger changes in symptoms over time, compared to those who did not have elevated anxiety to start with. This is an important consideration for future open access programs, as methods for evaluating outcome data must be relevant to the client population and the level of change expected. |
| Intervene with patients/consumers to enhance uptake and adherence  Audit and provide feedback | Strategies were also directly targeted at consumers to audit the program, provide feedback to inform refinements and to develop strategies for enhancing uptake and engagement with the program. These included:   - Eliciting feedback from end-users during program sessions to gauge satisfaction, likes, dislikes - Conducting cohort wide detailed satisfaction and feedback survey implemented for 3 months in 2015 to capture specific program feedback from early adopters. Data from the survey informed program upgrades as well as ongoing promotion efforts and implementation outcomes (see Aim 2 results). - Ongoing evaluation of platform metrics (engagement) to inform refinements. |
| ***Sustainability Phase (2017-2024)*** | |
| Use mass media  Distribute educational materials | Promotion and awareness building strategies included   - ongoing social media and internet dissemination - ongoing media releases at key points of program (eg, 10,000 users, 5 years) - further integration of the program weblink into relevant stakeholders and services (eg, beyondblue, youth beyondblue, ReachOut, Beacon, eMHprac, Emerging Minds and Healthdirect) - academic outreach and promotion. |
| Inform opinion leaders  Involve executive boards | During the sustainability phase, with the evidence established, the program was endorsed by the Royal Australian and New Zealand College of Psychiatrists and the Australian Psychological Society in their Clinical Practice Guidelines for anxiety disorders. |

#### Examples of the Implementation Strategies in Practice

We adopted several implementation strategies in line with Powell’s recommended effective strategies to promote program awareness, user and professional uptake, and positive user engagement. For example, strategies employed to enhance program awareness and engagement with the program included attendance at conferences targeted towards educational and mental health practitioners, delivering training guides and seminars to education and health professionals in local regions (a collaborative network), and social media and mass media campaigns championed by a national peak mental health body (*beyondblue).* Strategies were employed to promote uptake by regional families, including targeted distribution of promotional and educational material to professionals working in rural and regional areas, presentations at local, community and academic events in regional areas (eg, Rural and Remote Mental Health Conference, Caring for Country Kids), and conducting educational outreach visits to provide training to rural education and health practitioners. During the program’s implementation, over 30% of user registrations came from young people living in regional and rural areas. Given the program targeted school-age children, the education context was considered an important setting through which to encourage professionals to refer into and young people to uptake the program. Given the universal requirement for children to attend schooling and the first-hand knowledge school professionals have about the child’s behaviors and potential anxiety, the school context can be ideal for problem identification. Promotion strategies in school and educational contexts included targeted campaigns to promote awareness and knowledge of the program (email flashes, training and resource distribution), its uses and referral processes among school-based professionals and parents. During the program’s implementation 45% of program participants were referred by school professionals.

In terms of enhancing engagement, communication and support strategies for technical assistance were important in addressing potential problems with engagement. Whilst individual consumers rarely contacted the project team for therapeutic support, regular queries regarding general program and technical matters were received as were queries from health and educational professionals about how to use the program with their clients/students in various contexts. Ongoing communication and support were therefore essential to ensure that users could overcome obstacles, especially given there were no restrictions on use and no personalized monitoring of progress. Communication strategies included provision of detailed information on the website landing pages to enable users to address obstacles immediately (eg, FAQs, common technical problems, professional guide for download), as well as the provision of timely response to individual queries to maximize the chances of the user returning to the program. Audit and feedback strategies direct to consumers were also important in enhancing engagement. In the early stages of implementation, we identified a relatively low rate of commencement of program sessions following initial contact with the site. We determined that there were a proportion of users who registered for the program as a means of exploring the site rather than commencing the program. We subsequently introduced a guest access feature, whereby people could engage in a 20-minute trial before registering for the program. This successfully reduced the rate of users registering but not commencing the program. Throughout the course of the project, in response to consumer feedback, we also increased the frequency of email reminders and enhanced email content, to promote further engagement with the program.

**Strategy 1: Establishing Program Governance**

*Governance structures and responsibilities*

***Advisory Panel***

- Established to oversee project and comprised of:
- BRAVE Research Investigator team
- 2 x external, international experts in digital mental health and youth mental health
- representatives from funding body
- 2 x youth members

***Project Management Group***

- established to implement day-to-day program management
- comprised of BRAVE chief team member and research assistants
- program and promotion management meetings to plan promotional strategies and monitor progress; held fortnightly for first 2 years, then monthly in last year of project, including research team, and funder representatives
- established and monitored specific email and phone contacts for program participants and professionals
- continually tracked website and program usage to inform program refinements
- supported stakeholders in the promotion of the program to schools, clinicians and communities

**Strategy 2.** **Establish technical and implementation readiness of platform**

Key strategies for ensuring technical and implementation readiness:

- scoping of technical and interface changes required to program based on user and therapist feedback obtained in delivery of BRAVE-ONLINE Therapist-Assisted
- development of new program interface and age-appropriate landing pages following user experience principles
- refinement of interface following feedback from advisory panel (funder, expert panel members and youth representatives)
- piloting refined interface with consumers (rapid prototype/rapid feedback) to ensure effective design (look and feel) for targeted audiences (child or teen)
- refinement of interface to address consumer feedback (addition of trial video demonstrations)
- integration of existing BRAVE-ONLINE program into new interface
- expansion of key treatment techniques to enhance usability without therapist assistance
- interactive exposure hierarchy resource (Brave Ladder) developed
- relaxation room and user-informed relaxation resources developed
- resource folder developed including quick-grab handouts/tips
- enhancement of administrative back end to facilitate recording of program metrics (usage, engagement, reporting)
- enhancement of program stability on tablet devices
- technical testing of the program across devices and platforms.
- User testing during ‘production testing’ with 200 invited users (previous BRAVE-ONLINE research participants, young people with anxiety)
- Soft open launch of the program

**Strategy 3 – Establish a Promotion and Dissemination Plan**

a) *Development of age-appropriate promotional resources*

Targeted promotional resources were designed and developed by graphic and media designers at the University and beyondblue. These included age-appropriate flyers (parent-focused), postcards (child- and parent-focused), and wallet cards (adolescent-focused) that were distributed through the promotion plan and made freely available for downloading and ordering on the BRAVE website. Additional promotional resources were developed for stakeholders, including printable posters, newsletter inserts, and a Guide for Mental Health and School Professionals.

| DL Flyer for parents; front and back | | 10,000 hard copies distributed in Establishment Phase |
| --- | --- | --- |
| A5 postcard for parents/children; front and back   | | 10,000 hard copies distributed in Establishment Phase |
| Wallet size cards for adolescents; front and back | | 5000 hard copies distributed in Establishment Phase |
| Printable posters | Newsletter inserts | Professionals Guide |

b) *Social media and internet dissemination*

- The program was launched with a Facebook post by beyondblue that received 13,024 ‘likes’, ‘comments’ or ‘shares’, and reached 347,648 people.
- A promotional video disseminated through beyondblue and youthbeyondblue websites. BRAVE Program listed as key treatment recommendation in Brain Quiz administered through youthbeyondblue.
- Brave Program linked as treatment program recommendation through external organisations (eMHPrac, Beacon, ReachOut)
- Program promoted on Australian health or mental health-related websites; MindHealthConnect, The Kids are all right, Raising Children’s Network, Better Health Channel, Virtual Medical Centre
- BRAVE Program promoted through the e-Mental Health in Practice (eMHPrac) project delivering digital mental health workshops nationwide
- Twitter, Facebook and Instagram posts were staggered throughout the project period (posted by beyondblue and the BRAVE Team) to celebrate registration milestones (ie, 5000, 10,000 participants) as well as BRAVE team achievements (eg, national awards), linking into specific youth and anxiety-based events (eg, National Anxiety Week) or advising of attendance at events and conferences.
- National education wellbeing and training organization (Kids Matter) promoted BRAVE program in their e-newsletters within each state of Australia

c) *Media releases*

- Media releases by University and beyondblue at launch to local, state and national targets. This resulted in many newspapers, online news and magazine stories, including articles by Fairfax Media, the Sydney Telegraph and radio interviews (eg, Radio ABC South East).
- Television Interviews: The BRAVE Program was featured in a national news documentary about youth anxiety following the release of the National Survey of Youth Mental Health and Wellbeing.
- The program was featured for additional TV segments, print and online stories timed with achievements of the team (eg, winning national awards).

d) *Linking to stakeholders and services*

- Webinar presentation to over 300 mental health workers including psychologists, social workers, OT’s, other allied health, counsellors and peer workers as part of the e Mental Health in Practice (eMHPrac) program.
- Ongoing promotion through national education organization (KidsMatter and MindMatters programs)
- Promotion to schools and school guidance counsellors via email flashes – Informational emails along with promotional resources emailed to approximately 7000 state schools, inviting school professionals to use the Guide for School Professionals with their students.
- Program information distributed to General Practitioners through Rural Doctors Association of Australia, as well as the network of GP Super Clinics across Australia. Information about BRAVE Self-Help is also now included in the eMHPrac Project – a guide of e-mental health resources created for primary care providers.
- Promotional messages were sent to: MindMatters and SensAbility databases, Anxiety Disorders Alliance, ADAVIC Anxiety Disorder Association Victoria, Headspace, SANE, ReachOut, Child and Adolescent Mental Health Service (CAMHS), Child and Youth Mental Health Services (CYMHS), Pathways Health and Research Centre, The institute for Child and Youth Mental Health, Practice Pules (email from Medicare to GPs).
- Informational and promotional emails were sent to over 900 practicing psychologists whose target client base were young people with anxiety.
- Hardcopy distribution of resources, to:
  - Headspace centers across Australia.
  - Schools –over 5000 promotional flyers and postcards were distributed to interested schools.
  - Local QLD child health and mental health centers
  - Brave Self-Help Program promotions continued via the University of Southern Queensland’s Discovery Day for Years 7 and 8 school students, Careers Day for students in Years 9 and 10, and Student Experience Day for students in Years 11 and 12. Resource packs were provided to school professionals and beyondblue promotion cards were provided to students.

e) *Academic Promotion*

The BRAVE team was active in promoting the program in both academic and community-based events, conferences and workshops. Typically, this was also accompanied by the distribution of resources to delegates. A summary of these efforts is provided below:

- The BRAVE Self-Help program was promoted annually by beyondblue and the BRAVE Team at the Positive Schools conferences across the country (Brisbane, Perth, Sydney and Melbourne) (2014, 2015, 2016);
- Digital Rural Futures Conference in 2014, an event focused on the opportunities of Digital Resources to support the wellbeing of Regional and Rural communities;
- Preliminary results and a description of the program were presented at the 6th Annual Rural and Remote Mental Health Symposium in Albury in 2014;
- A ‘How to use BRAVE’ presentation was given at the 2015 Australian Psychologists and Counsellors in Schools Conference. This conference was targeted towards clinicians and counsellors working within the school sector;
- Presentations as part of Mental Health Week at four Queensland Health and Education Queensland staff training events;
- beyondblue provided promotional support at The Teen Expo in Melbourne, which saw close to 10,000 school aged children, parents and teachers attend. Information was also disseminated through other beyondblue Roadshow activities;
- Results of the program presented at the Australian Rural and Remote Mental Health conference in 2015;
- Oral presentation and information display at the Youth Health 2015 conference in Melbourne, including an exhibition at the Youth Forum, attended by 100 young people. The Youth Health conference was attended by health professionals from across Australia;
- Poster presentation at the Child Health Queensland Research Day in December 2015 at the Lady Cilento Children’s Hospital;
- Oral presentation outlining BRAVE-Self Help at the Gold Coast Health and Medical Research Conference (December 2015);
- Presentation at the Caring for Country Kids Conference in Alice Springs in April 2016, to healthcare professionals in regional, rural and remote Australia;
- Presentation of Brave Self-Help as a national dissemination strategy at 8th World Congress of Behavioral and Cognitive Therapies (Melbourne, 2016) to mental health practitioners, researchers and policy specialists from around the world;
- Presentations at the Institute of Professional Learning at the University of Sunshine Coast, in association with the e-Mental Health in Practice (eMHPrac) program. These presentations were delivered to 45 primary and secondary School Guidance Officers from the North Coast Region in 2016;
- A presentation at the Therapists, Nurses and Teachers Conference, (Brisbane) 2016, delivered to practicing therapists, nurses and teachers.

**Strategy 4 – Iterative, data-informed refinement**

Key data-informed refinements:

- monitoring and upgrading of program processing speed and memory capacity to manage software and hardware upgrades
- improving the program’s ability to capture user data, and to provide more detailed information on who was using the program and how it was being used
- adding questions to the registration process asking users how they found the program to assess success of promotion strategies
- improving the specificity of data exportation to support a fast-growing user database and allow for improved reporting
- development of a new ‘survey maker’ system that allowed the inclusion of additional, flexible items to existing hard-coded surveys. This was implemented to provide ability to answer clinical questions arising from project findings (eg, to gather data on user motivation to determine its relationship with program engagement)
- development of a new ‘frequently asked questions’ and ‘order promotional resources’ section for professionals in response to feedback from education and mental health professionals. This also allowed direct ordering of promotional materials in response to the increasing number of program enquiries.
- live analysis of satisfaction data to detect any themes relating to treatment engagement and program completion
