# Supplementary material for: 10 Years of BRAVE Self-Help Service Delivery for Child and Adolescent Anxiety in Australia: Open Effectiveness-Implementation Trial
Source: J Med Internet Res. 2026 Oct 5;28:e97810. doi: 10.2196/97810 (PMC13638004; doi:10.2196/97810)
Supplement: Supplementary file 2 [file jmir-v28-e97810-s002.doc]

Implementation Outcomes.

Trends in service utilisation and presenting anxiety severity, per year by user characteristics.

**User type**

**Figure S1.** New registrations per year by user type.	**Figure S2.** Presenting anxiety severity per year by user type.

**Gender**

**Figure S3.** New registrations per year by user gender.	**Figure S4.** Presenting anxiety severity per year by user gender.

**Remoteness**

**Figure S5.** New registrations per year by user remoteness.	**Figure S6.** Presenting anxiety severity per year by user remoteness.

**Table S1. Changes in presenting anxiety severity for all users including those with elevated anxiety from January 1 2015 to December 31 2024^a^.**

|  | |  | Baseline CAS-8^b^ | Final CAS-8 |  |  |  |
| --- | --- | --- | --- | --- | --- | --- | --- |
|  | | N | Mean (SD) | Mean (SD) | *t* test (df) | *P* value | Cohen *d* |
| All participants | | 31,578 | 11.16 (4.84) | 9.75 (5.39) | 58.26 (31,577) | <.001 | 0.33 |
| **Presenting anxiety severity** | |  |  |  |  |  |  |
|  | Elevated anxiety | 15,989 | 14.94 (3.28) | 12.43 (5.23) | 68.35(15,988) | <.001 | 0.54 |
|  | Non-elevated anxiety | 15,503 | 7.28 (2.59) | 6.99 (3.99) | 9.86 (15,502) | <.001 | 0.08 |

^a^ paired samples *t* test for changes in presenting anxiety severity throughout program participation, irrespective of the number of sessions completed (baseline anxiety to final anxiety scores, defined in analytic strategies).

^b^Children’s Anxiety Scale 8-item.

**Table S2. Mixed modelling for baseline to final scores on the CAS-8 measure for participants with elevated and non-elevated anxiety at baseline.**

|  | | **Unstandardized β coefficient** | SE | *P* value | 95% CI |
| --- | --- | --- | --- | --- | --- |
| **Baseline anxiety level** | |  |  |  |  |
|  | Elevated anxiety | –2.57 | 0.03 | <.001 | –2.64, –2.51 |
|  | Non-elevated anxiety | –0.28 | 0.03 | <.001 | –0.33, –0.23 |

**Figure S7.** Changes in the mean anxiety scores from a user’s baseline to final CAS-8 score over 10 years for users with elevated anxiety.

**Table S3. Proportions of participants demonstrating clinically meaningful change from baseline to final recorded session.**

|  | Participants Initially Elevated | | | Participants Initially Clinical | | |
| --- | --- | --- | --- | --- | --- | --- |
| User Program | N | Remained Elevated, n (%) | Crossed Elevated Threshold, n (%) | N | Remained Clinical, n (%) | Crossed Clinical Threshold, n (%) |
| Child | 4036 | 1455 (36.1) | 2581 (63.9) | 3842 | 1680 (43.7) | 2162 (56.3) |
| Adolescent | 2942 | 1458 (49.6) | 1484 (50.4) | 3926 | 2374 (60.5) | 1552 (39.5) |

**Table S4. Proportions of participants presenting with clinical levels of anxiety demonstrating clinically meaningful change into elevated and non-elevated levels from baseline to final recorded session.**

| User Program | N | Remained Clinical, n (%) | Reduced to Elevated, n (%) | Reduced to non-elevated, n (%) |
| --- | --- | --- | --- | --- |
| Child | 3842 | 1680 (43.7) | 885 (23) | 1277 (33.2) |
| Adolescent | 3926 | 2374 (60.5) | 916 (23.3) | 636 (16.2) |

**Table S5. Proportions of participants presenting with non-elevated levels of anxiety demonstrating clinically meaningful change into elevated and clinical levels from baseline to final recorded session.**

| User Program | N | Remained Non-Elevated, n (%) | Increased to Elevated, n (%) | Increased to Clinical, n (%) |
| --- | --- | --- | --- | --- |
| Child | 9942 | 8591 (86.4) | 964 (9.7) | 387 (3.9) |
| Adolescent | 5561 | 4848 (87.2) | 534 (9.6) | 179 (3.2) |
