# Supplementary material for: 10 Years of BRAVE Self-Help Service Delivery for Child and Adolescent Anxiety in Australia: Open Effectiveness-Implementation Trial
Source: J Med Internet Res. 2026 Oct 5;28:e97810. doi: 10.2196/97810 (PMC13638004; doi:10.2196/97810)
Supplement: Supplementary file 3 [file jmir-v28-e97810-s003.doc]

**Multimedia Appendix 3.** Feedback Survey with Young People and School Professionals.

**Background:** The BRAVE Feedback Survey was created for the purpose of evaluating participant satisfaction with the BRAVE Program, to investigate adherence rates to the program, and reasons that may contribute to failure to complete the program. Feedback surveys for both the child and teen programs were open for a period of 3 months during the second half of 2015. Participants who were already registered users of the program and who logged into the program during this time period were invited to complete the survey via a pop-up message upon login.

**Survey with Young People**

**Participants:** In total, 361 child program related participants and 273 teen program related participants (incorporating both child/teen and parent participants) commenced the survey. Given that the survey asked questions about how the participant used the program, participants were only included in the analyses if they had completed 1 or more sessions. Data from a participant was considered useable if 20% or more of the survey was completed (*N*=241 for child participants, and *N*=172 for teen participants).

*Age and Gender:*

**Figure S1.** Child participant gender breakdown. **Figure S2.** Teen participant gender breakdown.

**Figure S3.** Mean age of feedback participants.

*Sessions Completed:*

**Figure S4.** Child sessions completed.	**Figure S5.** Teen sessions completed.

*Support with Program:*

**Figure S6.** Proportion of participants completing the program with or without support.

*Reasons for starting BRAVE:*

**Figure S7.** Participant reasons for beginning the BRAVE program.

*Participant expectations of BRAVE:*

**Figure S8.** Participant expectations of the BRAVE program.

*Were participant expectations met:*

**Figure S9.** Proportion of child participants who reported their expectations of the BRAVE program were met.

**Figure S10.** Proportion of teen participants who reported their expectations of the BRAVE program were met.

The majority of participants were satisfied that the program met their needs; approximately 80% of child participants and 79% of teen participants. When asked what they had learnt, child participants indicated learning specific skills and strategies (24.88%) and normalization of their experience with anxiety, and increased knowledge and understanding (24.41%), along with improvement in their symptoms & confidence (14.08%) as key reasons for their satisfaction with the program.

Teen participants (64.71% response rate) noted improvement in their symptoms & confidence (21.59%), and an increase in the normalization of their experience with anxiety, and increased knowledge and understanding (13.64%) as well as learning specific strategies to help with managing their anxiety (11.36%).

*Reasons for non-adherence:*

- a reduction in symptoms may cause individuals to determine that they do not need to continue with the program (50.87% child and 38.94% teen).
- Boredom or lack of motivation (10.40% child, 19.47% teen).
- Being overwhelmed (3.54% teen)
- increased anxiety (0.88% teen)
- finding the program too confronting/challenging (1.77% teen)
- time constraints (10.40% child and 7.08% teen).
- the length of sessions/program (3.47% child, 8.85% teen)
- feeling that the program was not helping (8.09% child, 10.62% teen)
- technical issues (2.31% child)
- the requirement to complete homework (1.16% child)
- seeking other help (3.54% teen)
- embarrassment from others finding out that they were completing the program (1.77%)

*What They Liked:*

**Figure S11.** What child participants liked about the BRAVE program.

**Figure S12.** What teen participants liked about the BRAVE program.

*Most helpful components of BRAVE:*

**Figure S13.** The most helpful aspects of BRAVE (child participants).

**Figure S14.** The most helpful aspects of BRAVE (teen participants).

*What components would be most helpful if visiting BRAVE only once:*

- information about reducing anxiety (34% child, 26% teen).
- Thought (cognitive) management (25% child, 19% teen).

**Feedback Survey with School Professionals Feedback**

**Background:** This survey was conducted with school professionals who had previously expressed interest in the program or requested resources to gather information regarding how BRAVE Self-Help is being used in school settings. This was done to inform the development of an implementation guide for school professionals, which provides background information and practical suggestions for those interested in using the resource with their students. The survey was anonymous and included open questions for professionals to provide feedback. Professionals from some schools responded together, and we do not have descriptive information about the participant sample.

**Descriptive summary of feedback received:**

*How is the program being used?* The overall response from school professionals was very positive. In the school setting, the program was reported as commonly being used in a small-group format, where young people work through the program either individually or together (eg, on a smart board), with the assistance of a facilitator. Those acting as ‘facilitators’ assisting the young people to work through BRAVE include School Psychologists, Learning and Support Teachers, School Chaplains and School Nurses. Other uses of the program include one-on-one participation with the assistance of a staff member, having BRAVE Self-Help available in a ‘chill out’ room at lunch, working through the program in a whole class setting and recommending students and parents to work through the program at home.

*How was the program helpful?* Key ways in which the program was viewed as helpful included:

- introducing the ‘BRAVE language’ to young people as way to understand and communicate their thoughts and feelings at school and with their parents.
- Anecdotal reports of reduced anxiety levels amongst participating students
- Providing clear strategies to implement
- Improved communication with school professionals

*Quotes from School Professionals:*

*“I had a parent in the primary school say they had done the program with their child and it had changed both of their lives dramatically.  They both had learnt things together and were able to put in place things to help the child.  They had the same language to talk about what was going on so were able to tackle anxious thoughts and occasions and have lots of victories together”*

*“In all cases anxiety has improved. Students have less problems and are more relaxed. They start to use the breathing/relaxation and to feedback their improvements.  I find it is a better outcome for each session if the student can talk through it with us”*

*“There is so much that has worked well! I am finding that working through BRAVE when children are highly anxious has helped them calm down and activate helpful thoughts. The children are all using the language from the program and they all feel very comfortable coming to me and discussing any issues they are having.”*

*“They are very good at identifying their emotions and noticing when they are getting anxious - this was an issue before starting Brave.”*

*“I've also put another student onto it and she's doing it at home and her mum is doing the parent version and they're both finding it helpful.”*

*“It is a massive benefit to our students. Thanks again”*
